# Pannexin-1 channel opening is critical for COVID-19 pathogenesis

**DOI:** 10.1016/j.isci.2021.103478

**Published:** 2021-11-19

**Authors:** Ross Luu, Silvana Valdebenito, Eliana Scemes, Antonio Cibelli, David C. Spray, Maximiliano Rovegno, Juan Tichauer, Andrea Cottignies-Calamarte, Arielle Rosenberg, Calude Capron, Sandrine Belouzard, Jean Dubuisson, Djillali Annane, Geoffroy Lorin de la Grandmaison, Elisabeth Cramer-Bordé, Morgane Bomsel, Eliseo Eugenin

**Affiliations:** 1 Department of Neuroscience, Cell Biology, and Anatomy, University of Texas Medical Branch (UTMB), Research Building 17, 105 11th Street, Galveston, TX 77555, USA; 2 Department of Cell Biology & Anatomy, New York Medical College, Valhalla, NY, USA; 3 Dominick P. Purpura Department of Neuroscience & Department of Medicine (Cardiology), Albert Einstein College of Medicine, New York, NY 10461, USA; 4 Departamento de Medicina Intensiva, Facultad de Medicina, Pontificia Universidad Católica de Chile, Santiago, Chile; 5 Hôpital Cochin, Service de Virologie, Hôpital Cochin (AP-HP), Paris, France; 6 Service d’Hématologie Hôpital Ambroise Paré (AP-HP), Boulogne-Billancourt, France; 7 Virologie Moléculaire et Cellulaire des Coronavirus, Centre d'infection et d'immunité de Lille, Institut Pasteur de Lille, Université de Lille, CNRS, Inserm, CHRU, 59000 Lille, France; 8 Service des Maladies Infectieuses, Centre Hospitalier Universitaire Raymond Poincaré, AP-HP, Garches, France; 9 Intensive Care Unit, Raymond Poincaré Hospital (AP-HP), Paris, France; 10 Simone Veil School of Medicine, Université of Versailles, Versailles, France; 11 University Paris Saclay, Garches, France; 12 Department of Forensic Medicine and Pathology, Versailles Saint-Quentin Université, AP-HP, Raymond Poincaré Hospital, Garches, France; 13 University of Versailles Saint Quentin en Yveline, Versailles, France; 14 Mucosal Entry of HIV and Mucosal Immunity, Institut Cochin, Université de Paris, Paris, France; 15 INSERM U1016, Paris, France

**Keywords:** Cell biology, Molecular physiology, Virology

## Abstract

Severe acute respiratory syndrome coronavirus 2 (SARS-CoV-2) rapidly rampaged worldwide, causing a pandemic of coronavirus disease (COVID -19), but the biology of SARS-CoV-2 remains under investigation. We demonstrate that both SARS-CoV-2 spike protein and human coronavirus 229E (hCoV-229E) or its purified S protein, one of the main viruses responsible for the common cold*,* induce the transient opening of Pannexin-1 (Panx-1) channels in human lung epithelial cells. However, the Panx-1 channel opening induced by SARS-CoV-2 is greater and more prolonged than hCoV-229E/S protein, resulting in an enhanced ATP, PGE_2_, and IL-1β release. Analysis of lung lavages and tissues indicate that Panx-1 mRNA expression is associated with increased ATP, PGE_2_, and IL-1β levels. Panx-1 channel opening induced by SARS-CoV-2 spike protein is angiotensin-converting enzyme 2 (ACE-2), endocytosis, and furin dependent. Overall, we demonstrated that Panx-1 channel is a critical contributor to SARS-CoV-2 infection and should be considered as an alternative therapy.

## Highlights

• Pannexin-1 (Panx-1) is a critical player in COVID-19 lung pathogenesis
• SARS-CoV-2 Panx-1 channel opening is ACE-2/furin/endocytosis dependent
• SARS-CoV-2-associated Panx-1 channel opening induces inflammation
• Blocking Panx-1 channels reduces viral replication and inflammation

## Graphical abstract

**Figure:**
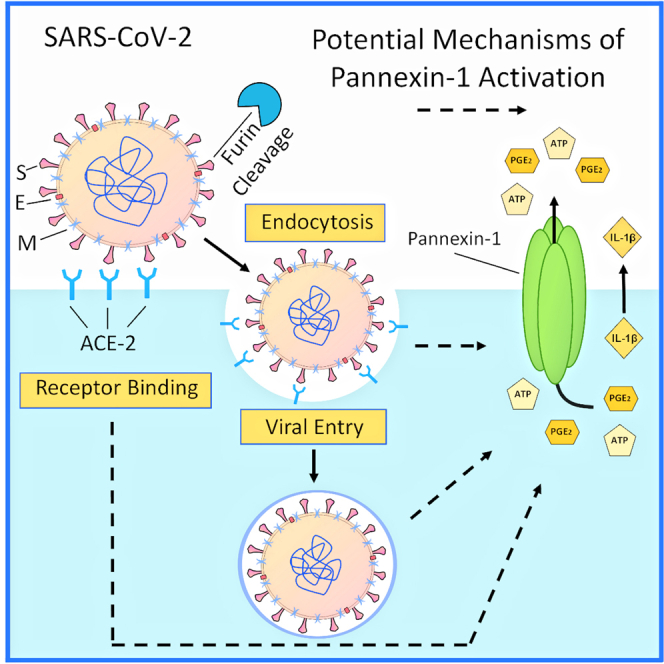

## Introduction

Viruses have evolved to use host-encoded proteins to facilitate infection, replication, and associated pathogenesis (Konig et al., 2008; Krupovic et al., 2018; Pillay, 2020; Walsh and Mohr, 2011; Zhou et al., 2017). In severe acute respiratory syndrome coronavirus 2 (SARS-CoV-2), several host proteins have been described, but far more research is required to fully understand the mechanism of viral entry, replication, and pathogenesis, as well as vaccine evaluation (Bao et al., 2020; Hartenian et al., 2020; Tay et al., 2020; V'Kovski et al., 2021; Yuki et al., 2020). SARS-CoV-2 is a positive-sense, single-stranded RNA-based betacoronavirus with an envelope decorated with a notable surface glycoprotein, the spike (S) protein. Viral entry occurs through the interaction of the S protein with the cellular receptor, angiotensin-converting enzyme 2 (ACE-2), and is facilitated by the processing of the S protein by proteases such as trypsin/furin (Shang et al., 2020; Wu et al., 2020), the transmembrane serine protease 2 (TMPRSS2) (Hoffmann et al., 2020a, 2020b), and endosomal cathepsins (Shang et al., 2020). The trypsin/furin preactivation of the S protein is a key difference among coronaviruses, resulting in an increased binding affinity to its receptor and a widespread infectious cycle (Shang et al., 2020). However, additional host factors contributing to the viral cell cycle and associated inflammation are still under investigation. Our data demonstrated that SARS-CoV-2 uses Pannexin-1 (Panx-1) channels to mediate infection and associated inflammation.

Panx-1 is widely expressed and forms oligomeric plasma membrane channels (Baranova et al., 2004; Michalski et al., 2020; Penuela et al., 2013; Qu et al., 2020; Ruan et al., 2020; Spagnol et al., 2014; Wang and Liu, 2021). Panx-1 channels have unique characteristics; they contain one of the largest mammalian pores enabling the release of small ions, nucleotides, lipids, and small RNA into the extracellular space (MacVicar and Thompson, 2010; Pelegrin and Surprenant, 2006; Qu et al., 2011). In healthy conditions, Panx-1 channels are generally in a closed state. However, during disease conditions such as stress, cancer, neurodegeneration, and HIV, the channel becomes open, resulting in the release of multiple pro-inflammatory factors (Gajardo-Gomez et al., 2020; Orellana et al., 2011a; Penuela et al., 2014; Seror et al., 2011; Velasquez and Eugenin, 2014; Velasquez et al., 2016).

Our laboratory previously demonstrated that HIV induces Panx-1 channel opening to accelerate viral entry, replication, cell-to-cell spread, and inflammation (Gorska et al., 2021; Malik and Eugenin, 2019; Orellana et al., 2013; Valdebenito et al., 2018; Velasquez and Eugenin, 2014; Velasquez et al., 2016). Furthermore, we identified that ATP secreted through the Panx-1 channel pore activates purinergic receptors that initiate signaling, immune recruitment, and inflammation (Eugenin, 2014; Gajardo-Gomez et al., 2020; Gorska et al., 2021; Velasquez et al., 2020). Similar interactions between Panx-1 and purinergic receptors have been described in multiple lung functions such as blood pressure control, vasodilation/constriction, airway defense, and viral infection (Haywood et al., 2021; Ledderose et al., 2018; Martinez-Calle et al., 2018; Riteau et al., 2010; Swayne et al., 2020; Wirsching et al., 2020). In addition, an active role of Panx-1 channels has been described in mucin hypersecretion (Seminario-Vidal et al., 2011; van Heusden et al., 2021), hydrostatic pressure (Richter et al., 2014), and general lung inflammation (Adamson and Leitinger, 2014). More importantly, blocking Panx-1 channel opening reduces *Pseudomonas aeruginosa* infection and associated inflammation (Maier-Begandt et al., 2021; Wonnenberg et al., 2014), the deleterious effects of smoking (Baxter et al., 2014), cystic fibrosis (Higgins et al., 2015), lung-associated heart failure (Dahl et al., 2016), blood vessel compromise (Luo et al., 2017), ischemia-reperfusion (Kirby et al., 2021; Sharma et al., 2018), coagulation (Taylor et al., 2021), and ventilator-associated damage (Jia et al., 2018), suggesting a potential therapeutic approach for COVID-19. All these conditions are present and exacerbated in COVID-19 patients (Barnes et al., 2020; Calabrese et al., 2020; Gupta et al., 2020; Kempuraj et al., 2020; Lo et al., 2020), suggesting that preventing Panx-1 opening could reduce infection and associated inflammation. However, whether Panx-1 channels and ATP are involved in COVID-19 pathogenesis was unknown.

Here, we show that Panx-1 channels open in response to SARS-CoV-2 or hCoV-229E. Our data indicate that SARS-CoV-2 S protein opens Panx-1 channels aggressively and for a prolonged time compared with hCoV-229E or its purified S protein, suggesting a different opening mechanism. Furthermore, we identify that the SARS-CoV-2-induced Panx-1 opening is ACE-2, furin, and endocytosis dependent. In agreement, upon Panx-1 channel opening induced by SARS-CoV-2 S protein, ATP, PGE_2_, and IL-1β are released into the extracellular space. Lung tissue and nasal swab analysis indicate that Panx-1 mRNA and protein are increased in immune and lung cell populations, supporting the essential role of the Panx-1 channel in COVID-19 pathogenesis. *In vivo* analysis of lung lavage confirmed elevated ATP, PGE_2_, and IL-1β concentrations compared with other lung-related diseases such as chronic obstructive pulmonary disease (COPD), indicating a strong and acute physical response to SARS-CoV-2. We propose that blocking Panx-1 channels or associated purinergic signaling could prevent the devastating acute and long-term consequences of COVID-19 or other emerging coronaviruses.

## Results

### SARS-CoV-2 and hCoV-229E induced the opening of Panx-1 channels

Primary cultures of human lung epithelial cells were treated with S Protein (recombinant S protein of SARS-CoV-2, the agent of COVID-19, 1 μg/mL) or 229E (hCoV-229E, the common cold virus, 0.1 MOI or recombinant S protein, 1–5 μg/mL) to determine the Ethidium uptake rate (Etd, 5 μM), by live-cell imaging, as a measure of Panx-1 channel opening. The Etd dye only crosses the plasma membrane in healthy cells by passing through specific large channels such as connexin (Cx) hemichannels and Panx-1 channels. Therefore, as a function of time, its intracellular fluorescence reflects channel opening as determined by the treatment with specific channel blockers (Contreras et al., 2002; Orellana et al., 2011b; Sanchez et al., 2009).

**Figure 1:**
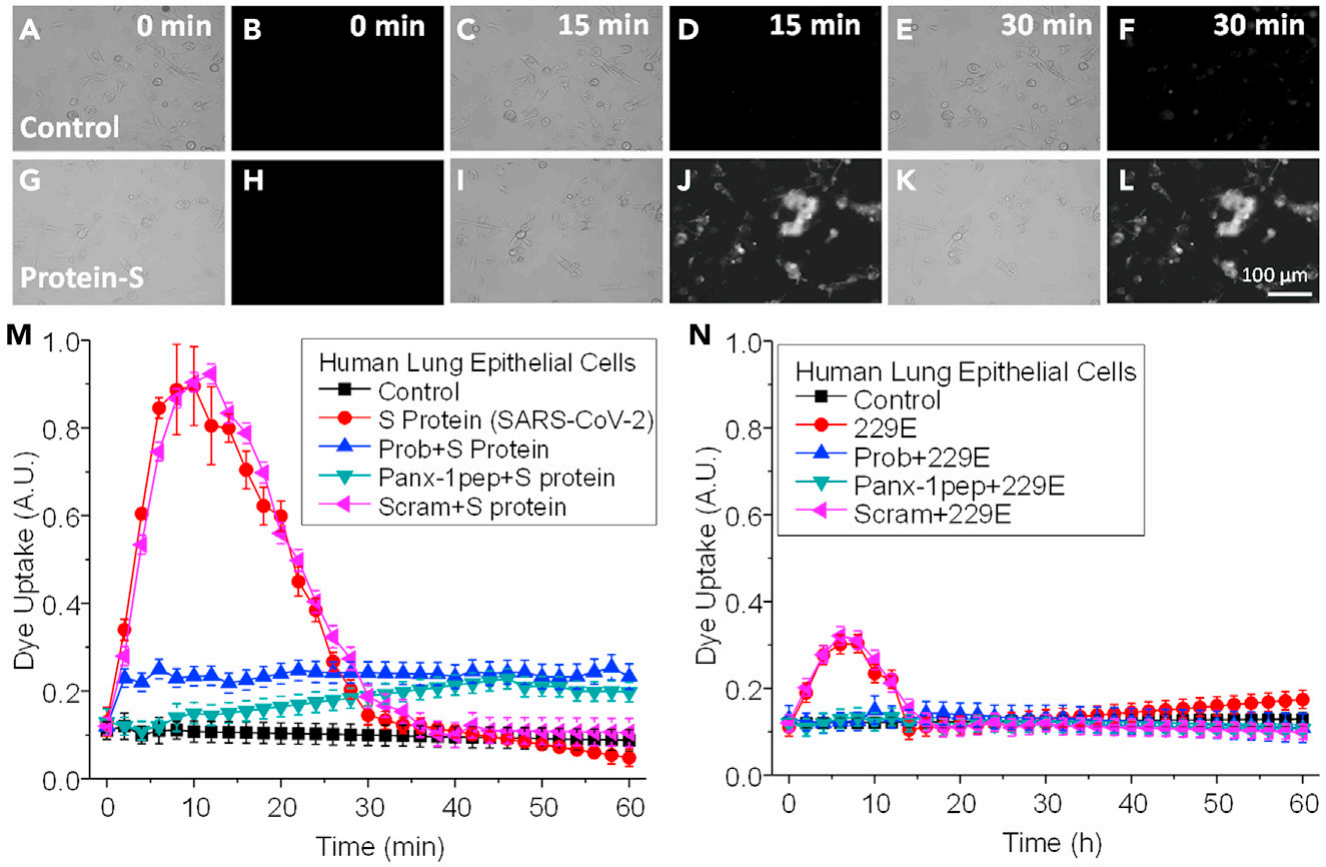
SARS-CoV-2 S protein induces Panx-1 channel opening in human lung primary epithelial cells (A–F) Representative snapshots of the time course of Etd uptake in airway epithelial cells for the control at 0, 15, and 30 min. Images shown for each time point are in duplicate with brightfield (left) and fluorescence (right) channels. (G–L) Representative snapshots of the time course after treatment with SARS-CoV-2 S protein (3.5 μg/mL) at 0, 15, and 30 min. Images shown for each time point are in duplicate with brightfield (left) and fluorescence (right) channels. (M) Quantification of the time course of Etd uptake rate from human lung epithelial cells under control conditions (black square) or after SARS-CoV-2 S protein treatment (red circle) for 0 to 60 min. Human lung epithelial cells were pretreated with Probenecid (Prob, blue upward triangle), Panx-1 mimetic peptide (Panx-1pep, green downward triangle), and the scrambled peptide (Scram, pink leftward triangle) for 10 min before the treatment with SARS-CoV-2 S protein. Probenecid and Pannexin-1 mimetic peptide prevented the Panx-1 channel opening induced by SARS-CoV-2 S protein. No significant differences were observed between the control, Prob + S Protein, and Panx-1pep + S protein-treated cells (p = 0.236, for all the points analyzed). No significant differences were observed between S protein and Scram treatments (p = 0.302, for all the points analyzed). For cells treated with S protein alone or S protein pretreated with the scrambled peptide, Scram, all the points from 4 to 24 min were significantly different from control conditions (p = 0.0002 compared to control conditions). Each value corresponds to the mean ± SD of the Etd intracellular intensity present in at least 20 cells per time point (n = 4). (N) Quantification of the time course of Etd uptake rate after treatment of human lung epithelial cells under control (black square) or after hCoV-229E treatment (red circle, 0.1 MOI shown) for 0 to 60 min. The Panx-1 channel opening induced by the 229E virus resulted in a significantly lower uptake rate than cells treated with SARS-CoV-2 S protein alone (p ≤ 1.67x10^−4^, for all the points analyzed). Human lung epithelial cells were pretreated with Probenecid (Prob, blue, upward triangle), Pannexin-1 mimetic peptide (Panx-1pep, green, downward triangle), and the scrambled peptide (Scram, pink leftward triangle) for 10 min before the treatment with hCoV-229E. For hCoV-229E and Scram+229E treatment, all time points, 4 to 12 min, were significantly different from control conditions (p = 0.001 as compared with control conditions). Each value corresponds to the mean ± SD of the Etd intracellular intensity present in at least 20 cells per time point (n = 3).

Nonspecific Etd uptake was not observed in the untreated or BSA-, furin-, or trypsin-treated (2.5–5 μg/mL) human primary lung epithelial cells (Figures 1A–1F corresponds to representative time points of untreated cells. Intracellular fluorescence rate quantification is indicated in Figures 1M or 1N, black lines, control-untreated). In contrast, treating human primary lung epithelial cells with S protein from SARS-CoV-2 (S protein, 1 μg/mL, Figures 1G–1L as representative examples) induced a strong and transient channel opening that returned to near baseline levels after ∼30 min (Figures 1G–1L, a representative example of live cell imaging treated with S protein. Figure 1M, red lines, quantification of the intracellular fluorescence rate of epithelial cells treated with S protein, p ≤ 0.00012, n = 3–7). Treatment of human primary lung epithelial cells with 229E (1–0.01 MOI, data shown in Figure 1N represent 0.1 MOI) or purified S protein (1–5 μg/mL) induced a smaller and more transient Panx-1 channel opening than SARS-CoV-2 S protein. The time course of Panx-1 opening induced by 229E reached a peak after 5–7 min posttreatment to return to baseline after 10 min (Figure 1N, red lines, 229E, p ≤ 1.67x10^−4^). Therefore, SARS-CoV-2 S protein and hCoV-229E or its S protein induced Panx-1 channel opening with a different time course and intensity. No cell death or loss was observed in all the cultures analyzed (data not shown).

To ensure whether Etd uptake induced by coronaviruses is solely a consequence of Panx-1 channel opening and no other large channels, we used Probenecid (Prob, 500 μM) and a Panx-1 blocking mimetic peptide (Panx-1 pep, 200 μM) to prevent Panx-1 channel opening in response to SARS-CoV-2 S protein or hCoV-229E (whole virus or its S protein). These treatments have previously been demonstrated to specifically block Panx-1 channels (Orellana et al., 2011a; Silverman et al., 2008, 2009; Velasquez et al., 2020). Preincubation (10 min) of human lung epithelial cells with Probenecid (Figures 1M and 1N, blue lines, Prob + S protein or Prob+229E, respectively) or the Panx-1 mimetic blocking peptide (Figures 1M and 1N, green lines, 200 μM Panx-1pep + S protein or Panx-1pep+229E, respectively) prevented the opening induced by the viral proteins (S protein, 2.5 μg/mL, or 229E, 0.1 MOI or its S protein, 1–5 μg/mL). Moreover, the preincubation of the epithelial cell cultures with the Panx-1 scrambled peptide (Scram, 200 μM) before treatment with S protein or 229E did not prevent the Etd uptake induced by the viral components (Figures 1M and 1N, pink lines, 200 μM Scram + S protein or Scram+229E, respectively, p ≤ 0.0002, n = 3–7, compared with control conditions). In contrast, Lanthanum (La^3+^), a general connexin hemichannel blocker, or Cx43^E2^, an antibody that blocks Cx43 hemichannels at several concentrations as described previously (Evans et al., 2006; Orellana et al., 2011b; Siller-Jackson et al., 2008), did not affect the S protein or 229E-induced Etd uptake rate (Figures S1A and S1B), suggesting that Cx43 hemichannels do not participate in the dye uptake process. Furthermore, no toxic or nonspecific effects of these blockers alone were detected as determined by trypan blue exclusion, TUNEL staining, or cell detachment (See Figure S1B). Overall, coronaviruses induced the Panx-1 channel opening on primary lung epithelial cells.

### Electrophysiology of primary human epithelial cells demonstrates that SARS-CoV-2 S protein in combination with ATP increased Panx-1 channel opening

**Figure 2:**
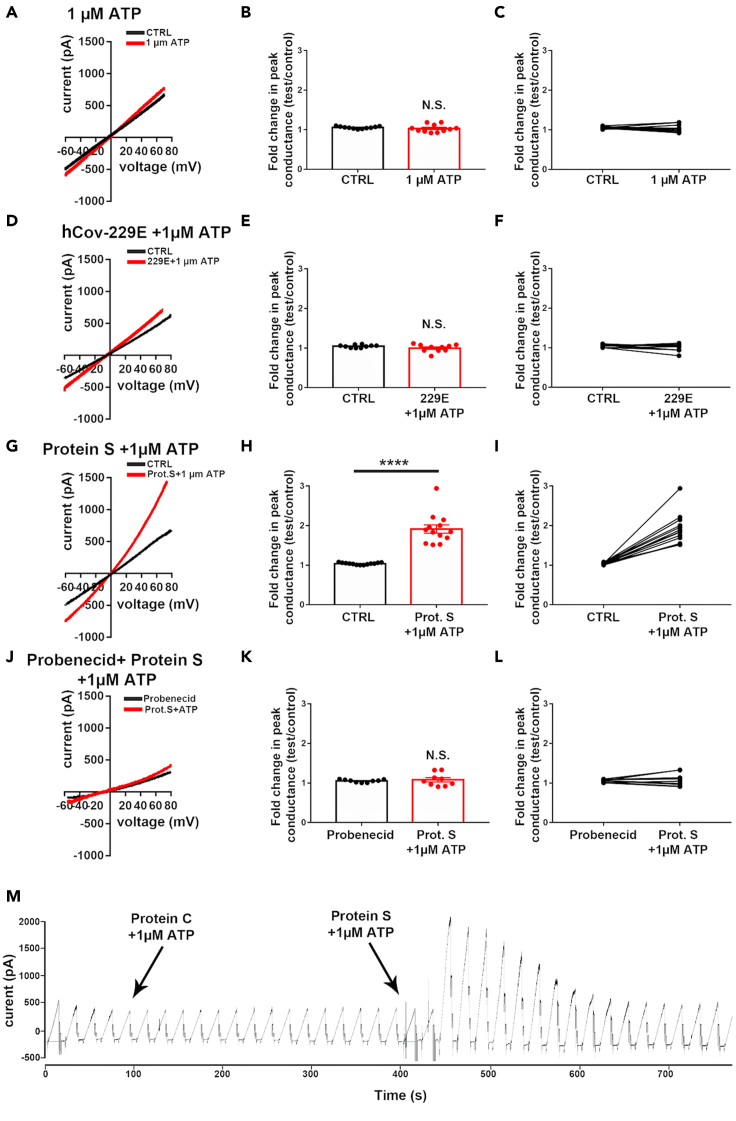
SARS-CoV-2 S protein induces the opening of Panx-1 channels (A–L) Electrophysiological recordings obtained from human epithelial cells in the absence (black) and presence (red) of (A–C) 1 μM ATP, (D–F) hCoV-229E heat-inactivated (229E, 1 μL/mL) plus 1 μM ATP, (G–I) SARS-CoV-2 S protein (Prot S, 2.5 μg/mL) plus 1 μM ATP, and (J–L) SARS-CoV-2 S protein plus 1 μM ATP in the presence of the Panx-1 channel blocker, Probenecid (Prob, 1.0 mM). (M) Example of currents recorded from an epithelial cell evoked by 12 s long voltage ramps (−70 mV to +70 mV) following the bath application of Prot S and 229E in the presence of ATP (arrows). Note that only SARS-CoV-2 S protein plus 1 μM ATP induced Panx-1 channel openings and that Prob prevented Panx-1 activation. (B, E, H, and K) show the mean ± SEM values of the fold changes in peak conductance measured from epithelial cells under the various conditions, and (C, F, I, and L) show the changes in peak conductance for each individual cell exposed to the experimental conditions. ∗∗∗∗p < 0.0001 (paired t test).

To expand our dye uptake analysis and ensure Panx-1 identity, we performed whole-cell patch-clamp recordings using human primary lung epithelial cells to examine the extent to which the Panx-1 channel is activated by SARS-CoV-2 S-protein (Figure 2). To that end, we measured changes in peak conductance induced by voltage ramps before and after adding either viral agent (in the presence of ATP) and normalized the conductance to those recorded in H-PBS alone. To verify that low doses of ATP (1 μM) do not induce Panx-1 channel opening but facilitate it, as occurs under several pathological conditions (Iglesias et al., 2008; Negoro et al., 2013; Silverman et al., 2009), fold changes in peak conductance were measured. As shown in Figures 2A–2C, addition of 1 μM ATP did not affect current amplitudes compared with control conditions (control: 1.05 ± 0.01 folds, ATP: 1.03 ± 0.03 folds; n = 11 cells, p = 0.47 paired t test). Treatment of primary human cultures of lung epithelial cells with 229E heat-inactivated (1 μg/mL) *plus* ATP (1 μM) resulted in nonsignificant Panx-1 channel opening (Figure 2D, hCoV-229E + ATP) as determined by the changes in peak conductance compared with untreated control conditions (Figures 2E and 2F, control: 1.05 ± 0.01 folds, hCoV-229E + ATP: 1.00 ± 0.03 folds, n = 10, paired t test, p = 0.341). In contrast, the application of SARS-CoV-2 S protein (1.0 μg/mL) plus ATP (1 μM) to lung epithelial cells resulted in a significant increase in the Panx-1 channel peak conductance, as indicated in the recordings (Figure 2G) and upon quantification of the fold changes in the peak of conductance (Figures 2H and 2I, control: 1.04 ± 0.01 folds, SARS-CoV-2 S protein + ATP: 1.91 ± 0.01 folds, n = 12, p < 0.0001 paired t test). To confirm that changes in conductance were related to Panx-1 currents, Probenecid (1.0 mM) was applied, and fold changes in peak conductance were measured in the presence of S protein (Figure 2J). Under this condition, no significant changes in peak conductance were recorded following application of S protein (Figures 2K and 2L, control + Prob: 1.05 ± 0.01 folds, Prob + Prot S: 1.08 ± 0.05 folds, n = 9, paired t test, p = 0.56). Thus, in accordance with the dye uptake data, our electrophysiological data confirmed that SARS-CoV-2 S protein induced a 2-fold increase in Panx-1 channel opening in the presence of ATP.

### Opening of Panx-1 channels in response to SARS-CoV-2 S protein or hCoV-229E or its S protein induces ATP, PGE_2_, and IL-1β release

Panx-1 channel opening has been associated with local and systemic inflammation as well as HIV entry into immune cells by a mechanism involving the local release of intracellular factors such as ATP, NAD^+^, prostaglandins, and other inflammatory lipids through the channel pore (Gajardo-Gomez et al., 2020; Qu et al., 2011; Velasquez and Eugenin, 2014; Velasquez et al., 2020). In addition, Panx-1 channel opening is indirectly associated with IL-1β processing and release (Lee et al., 2018; Pelegrin and Surprenant, 2006, 2009; Seil et al., 2010; Yang et al., 2019), suggesting that Panx-1 amplifies inflammation, but its role in COVID-19 pathogenesis is unknown.

**Figure 3:**
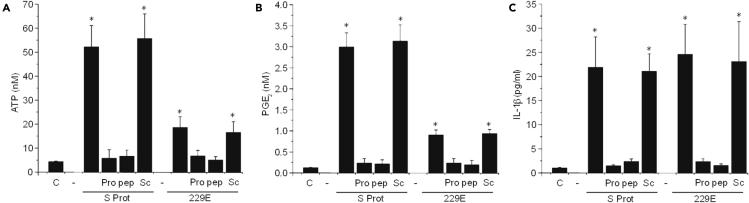
Opening Panx-1 channels in response to SARS-CoV-2 S protein or hCoV-229E-virus-induced ATP, PGE_2_, and IL-1β release Upon treating primary lung epithelial cells with SARS-CoV-2 S protein (1 μg/mL) or hCoV-229E (0.1 MOI), media was collected after 1, 6, 12, and 24 h posttreatment to quantify ATP, PGE_2_, and IL-1β. Data after 1 h posttreatment are represented. (A) Determination of ATP secretion from primary human airway epithelial cells for the control (C) and after S protein (S Prot) or 229E virus (229E) treatment in the presence or absence of Probenecid (Pro), Panx-1 mimetic peptide (pep), or the scrambled peptide (Sc). Relative to the control (C), SARS-CoV-2 S protein and hCoV-229E treatment induced an ATP secretion that was strongly Panx-1 dependent. Treatment with the S-protein-induced ATP secretion was ∼2.5-fold more effective than treatment with 229E (∗p ≤ 0.001, n = 4). Pretreatment with Panx-1 blockers, Probenecid, or the Panx-1 mimetic peptide, before treatment with the S Prot or 229E, did not result in significantly elevated concentrations of ATP. In addition, pretreatment with Scram before treatment with S protein or 229E resulted in elevated concentrations of ATP comparable with cells treated with S protein or 229E alone, respectively (∗p ≤ 0.001, n = 4). Each value corresponds to the mean ± SD (n = 4). (B) Determination of PGE_2_ secretion from primary human airway epithelial cells for the control (C) and after S protein (S Prot) or hCov-229E virus (229E) treatment in the presence or absence of Probenecid (Pro), Panx-1 mimetic peptide (pep), or the scrambled peptide (Sc). Relative to the control (C), SARS-CoV-2 S protein and hCoV-229E treatment induced a PGE_2_ secretion that was also strongly Panx-1 dependent. Treatment with the S-protein-induced PGE_2_ secretion was also ∼2.5-fold more effective than treatment with 229E (∗p ≤ 0.001, n = 4). Pretreatment with Panx-1 blockers, Probenecid, or the Panx-1 mimetic peptide before treatment with the S Prot or 229E did not result in significantly elevated concentrations of ATP. In addition, pretreatment with Scram before treatment with S protein or 229E resulted in elevated concentrations of PGE_2_ comparable with cells treated with S protein or 229E alone, respectively (∗p ≤ 0.001, n = 4). Each value corresponds to the mean ± SD (n = 4). (C) Determination of IL-1β secretion from primary human airway epithelial cells for the control (C) and after S protein (S Prot) or hCoV-229E virus (229E) treatment in the presence or absence of Probenecid (Pro), Panx-1 mimetic peptide (pep), or the scrambled peptide (Sc). Relative to the control (C), SARS-CoV-2 S protein- and hCoV-229E-treatment-induced IL-1β secretion was also strongly Panx-1 dependent. Treatment with the S-protein-induced IL-1β secretion was similar to treatment with 229E (∗p ≤ 0.001, n = 4). Pretreatment with Panx-1 blockers, Probenecid, or the Panx-1 mimetic peptide before treatment with the S Prot or 229E did not result in significantly elevated concentrations of ATP. In addition, pretreatment with Scram before treatment with S protein or 229E resulted in elevated concentrations of IL-1β comparable with cells treated with S protein or 229E alone, respectively (∗p ≤ 0.043, n = 3, relative to control conditions). Each value corresponds to the mean ± SD (n = 3). It should be noted that although IL-1β secretion is correlated with Panx-1 channel activity, it is an indirect measure of cellular activation.

Human primary lung epithelial cells were treated with SARS-CoV-2 S Protein or 229E (whole virus or its S protein) for 1, 6, 12, and 24 h, and media was collected to determine ATP, PGE_2_, and IL1β release by ELISA (Figures 3A–3C, respectively). In untreated control conditions (denoted by C in Figures 3A–3C), minimal baseline ATP, PGE_2_, and IL-1β release were observed (Figures 3A–3C, respectively). Treatment with SARS-CoV-2 S protein (S protein) or 229E virus induced ATP and PGE_2_ release, but treatment with SARS-CoV-2 S protein resulted in a greater than 2-fold increase in the secretion of both ATP and PGE_2_ than 229E (Figures 3A and 3B, respectively, ∗p ≤ 0.001, n = 4 independent experiments, only 1 h is shown, but data were consistent after 6, 12, and 24 h). These differences in ATP, PGE_2_, and IL-1β release correlated with the extent and degree of Panx-1 channel opening observed by dye uptake between both viral components (Figure 1). In contrast, IL-1β release was similar for both S protein and 229E (Figures 3C, S Prot or 229E, respectively), supporting that both viruses have different responses. Furthermore, we demonstrated that ATP, PGE_2_, and IL-1β release in response to S protein or 229E were Probenecid (Pro) and Panx-1 blocking mimetic peptide (pep) sensitive (Figures 3A–3C; Pro and pep, respectively), supporting the idea that their secretion is Panx-1 dependent. Preincubation with Probenecid (Pro) or Panx-1 blocking peptide (pep) did not affect the baseline release of ATP, PGE_2_, and IL-1β (data not shown). In addition, preincubation with Cx43 hemichannel blockers such as Lanthanum (La^3+^), a general connexin hemichannel blocker, or Cx43^E2^, an antibody that blocks Cx43 hemichannels, did not prevent the release of ATP, PGE_2_, and IL-1β (Figure S2). Furthermore, a scrambled Panx-1 peptide (Sc) did not prevent the Panx-1 channel opening and ATP/PGE_2_/IL-1β release induced by S protein or 229E (Figures 3A–3C, respectively). Overall, we identified that Panx-1 channel opening induced by SARS-CoV-2 recombinant S protein or hCoV-229E (whole virus or its S protein) resulted in inflammation.

### Panx-1 channel opening induced by SARS-CoV-2 S protein is dependent on ACE-2, endocytosis, and furin activity

To determine the mechanism of Panx-1 channel opening induced by SARS-CoV-2 S protein and hCoV-229E (whole virus or its S protein), as well as the differences observed in dye uptake/electrophysiology/secretion of intracellular inflammatory factors between both coronaviruses, we focused on the differences in the cell receptor, endocytosis, and furin dependency described for both viruses (Shang et al., 2020; Walls et al., 2020c).

To examine these mechanisms, we pretreated human primary lung epithelial cell cultures with a human recombinant protein ACE-2 (hrACE-2, 0.4 μg/mL) before treatment with SARS-CoV-2 S protein (S protein, 1μg/mL) or 229E (0.1 MOI) to assess the competitive binding of the virus to the endogenous ACE-2 protein as described (Lu and Sun, 2020). To prevent endocytosis, we used the endocytosis inhibitors ammonium chloride (NH_4_Cl) (50 mmol/L), bafilomycin-A1 (50 nmol/L), and chloroquine (100 μg/mL). NH_4_Cl and chloroquine are lysosomotropic agents that selectively accumulate in endocytic compartments and increase the endosomal pH. Bafilomycin-A1 (50 nmol/L) is a specific blocker of v-type H^+^-ATPase (Eugenin et al., 2008). Concentrations of these reagents were selected as such because they had been optimized to maximally inhibit the entry of avian leukosis virus into cells, a virus that requires endocytosis for infection as well as into smooth muscle cells (Eugenin et al., 2008). In addition, preincubation of S protein or 229E (whole virus or its S protein)-infected cells with furin (0.86 μg/mL) to promote the furin cleavage at the S1-S2 junction present in the SARS-CoV-2 S protein, but not present in other coronaviruses, was performed. SARS-CoV-2 S protein, therefore, has a furin cleavage site that strengthens the viral-host interaction through the ACE-2/S-protein interaction by 10-fold (Walls et al., 2020b) as compared with other coronaviruses, including hCoV-229E (Ge et al., 2013; Lau and Peiris, 2005), resulting in enhanced infectivity (Li et al., 2005).

**Figure 4:**
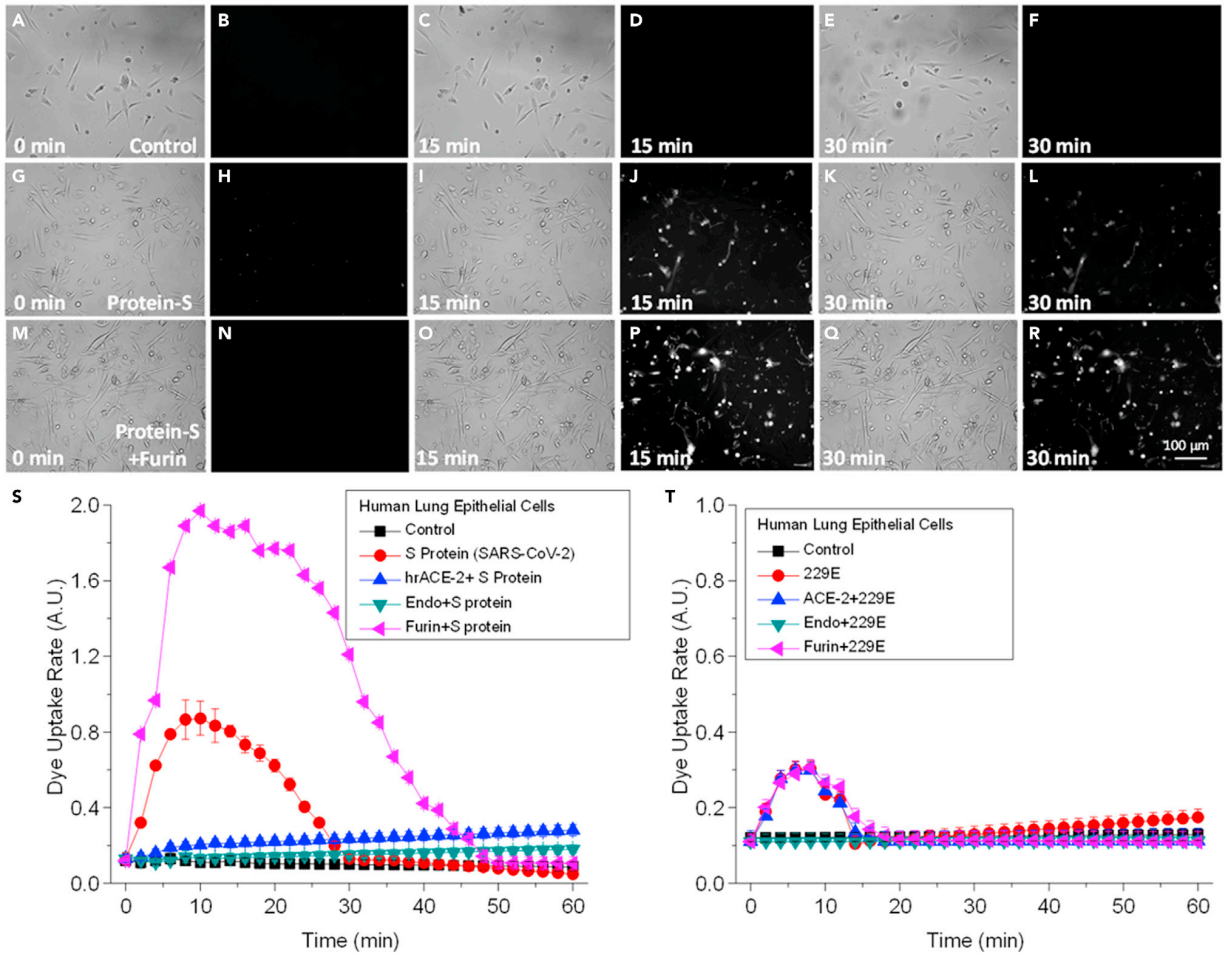
Panx-1 channel opening induced by SARS-CoV-2 is ACE-2, endocytosis, and furin dependent In contrast, Panx-1 channel opening in response to hCoV-229E is endocytosis dependent but ACE-2 and furin independent. (A–F) Representative snapshots of the time course of Etd uptake in airway epithelial cells for the untreated control at 0, 15, and 30 min. Images shown for each time point are in duplicate with brightfield (left) and fluorescence (right) channels. (G–L) Representative snapshots of the time course of Etd uptake in airway epithelial cells after treatment with S protein (1μg/mL) or 229E (0.1 MOI) or its purified S protein (5 μg/mL) at 0, 15, and 30 min. (M–R) Representative snapshots of the time course of Etd uptake in airway epithelial cells after pretreatment with furin (0.86 μg/mL) and then treatment with S protein (1μg/mL) or 229E (0.1 MOI) at 0, 15, and 30 min. (S) Quantification of the time course of Etd uptake for the airway epithelial cells that were untreated (black square) or treated with S protein alone (red circle) or S protein pretreated with human recombinant ACE-2 protein (0.4 μg/mL, blue upward triangle), endocytotic inhibitors (NH_4_Cl, 50 mmol/L; bafilomycin-A1, 50 nmol/L: and chloroquine, 100 μg/mL, green downward triangle), or furin (0.86 μg/mL, pink leftward triangle). Furin pretreatment of S protein resulted in the most significant uptake of Etd even relative to treatment with S protein alone (p ≤ 0.00025, n = 3, between 4 and 24 min for S protein and 4 and 42 min for furin-treated S protein compared with control conditions. Each value corresponds to the mean ± SD (n = 3). (T) Quantification of the time course of Etd uptake for the airway epithelial cells that were untreated (black square) or treated with the hCoV-229E (229E) alone (red circle) or 229E pretreated with human recombinant ACE-2 protein (0.4 μg/mL, blue upward triangle), endocytotic inhibitors (NH_4_Cl, 50 mmol/L; bafilomycin-A1, 50 nmol/L: and chloroquine, 100 μg/mL) (green downward triangle), or furin (0.86 μg/mL, pink leftward triangle). For this experiment, Panx-1 channel opening induced by hCoV-229E was only dependent on endocytosis but independent of ACE-2 and furin pathways (p ≤ 0.0012, n = 3) compared with control conditions. Denote the different scale between graphs S and T (2 folds increase in SARS-CoV-2 compared to 229E). Each value corresponds to the mean ± SD (n = 3).

For this experiment, we confirmed that Etd uptake in control-untreated conditions was minimal (Figures 4A–4F, at 0, 15, and 30 min. Dye uptake rate quantification is shown in Figures 4S and 4T, black, Control). Treatment with S protein or 229E induced Panx-1 opening as detected by Etd uptake (Figures 4G–4L, for S protein at 0, 15, and 30 min; data for 229E not shown. Dye uptake quantification is shown in Figures 4S and 4T, S protein and 229E, respectively in red). The preincubation of primary lung epithelial cells with hrACE-2 to compete for the binding of SARS-CoV-2 S-protein to the host ACE-2 prevented the opening of Panx-1 channels induced by S protein (Figure 4S, hrACE-2+S protein, blue). However, hrACE-2 did not affect the Panx-1 channel opening induced by 229E, suggesting a different opening mechanism than the S protein (Figure 4T, ACE-2+229E, blue). Pretreatment (10 min) of the human primary lung epithelial cells with the endocytosis inhibitors, ammonium chloride (NH_4_Cl), bafilomycin-A1, or chloroquine prevented the Panx-1 channel opening induced by the S protein and 229E virus, suggesting that endocytosis is essential to trigger Panx-1 channel opening (Figures 4S and 4T, green, Endo + S protein or Endo+229E, respectively).

SARS-CoV-2 S protein harbors a furin cleavage site at the S1/S2 boundary not present in other coronavirus S proteins, suggesting higher transmissibility and pathogenesis are associated with this change (Belouzard et al., 2009; Millet and Whittaker, 2015; Walls et al., 2020a). Thus, to determine whether furin processing of SARS-CoV-2 S protein participates in the Panx-1 channel opening, we preincubated the S protein and 229E with furin (0.86 μg/mL) for 10 min and then added them to the human lung primary epithelial cells to determine Panx-1 channel opening by dye uptake. Furin-treated S protein induced a greater and more prolonged Panx-1 channel opening than untreated S protein treatment (compare Figures 4G–4L, S protein versus Figures 4M–4R, S protein treated with furin. Fluorescence quantification indicates that furin treatment increased channel opening and the duration of opening by at least 2-fold. Note the difference in the Y axis between Figures 4S and 4T). However, furin treatment does not affect 229E or its S protein on Panx-1 channel opening (Figure 4T, pink, Furin+229E). The alterations to the original time course of Panx-1 channel opening induced by furin proteases were sensitive to Probenecid and Panx-1 blocking mimetic peptide (data not shown). No significant cell death or loss was observed during the time course analyzed in the presence of S-protein, blockers, or furin treatment (apoptosis remains under 5%).

Quantification of ATP, PGE_2_, and IL1β release 1 h after furin treatment of S protein indicates that furin increased ATP secretion (197.9 ± 36.98 nM, p = 0.0025, n = 4) and PGE_2_ (8.04 ± 2.09 nM, p = 0.0002, n = 4) but did not affect IL1β secretion (20.84 ± 6.99 pg/mL, p = 0.563, n = 4) compared with S protein without furin pretreatment. In contrast, furin treatment of 229E did not significantly affect the secretion of ATP, PGE_2_, and IL1β (compared with Figure 3). 229E (the whole virus or 1–5 μg/mL of S protein) treatment for 1 h induced an ATP (18.5 ± 7.78 nM), PGE_2_ (1.12 ± 0.21 nM), and IL1β (21.02 ± 6.08 pg/mL) release from human epithelial cells, suggesting a different mechanism of cell activation compared with SARS-CoV-2. Further, experiments with 299E S protein indicated that ATP secretion was comparable with the whole virus (16.34 ± 9.32 nM). The increase in MOI (10, 5, and 1) did not mimic the data obtained using SARS-CoV-2 S protein, indicating that protein concentration or viral titer do not account for the differences in the inflammatory profile. Furin, endocytic blockers, hrACE-2, or BSA alone, used as a control, did not affect the basal Panx-1 channel opening or ATP, PGE_2_, and IL1β secretion during the time course analyzed or triggered apoptosis (data not represented). Overall, our data demonstrate that SARS-CoV-2 has an inflammatory profile that is different from hCoV-229E.

### Panx-1 blockers prevented SARS-CoV-2 replication

SARS-CoV-2 viral infection of human lung epithelial cells was performed for 6 h (MOI 0.1) to enable viral entry, and subsequently, different treatments were used to examine SARS-CoV-2 replication at different time points, 12, 24, 48, and 72 h. Viral replication was determined using staining with the V-nCoV2019 sense RNAscope probe, an *in-situ* hybridization probe for detecting SARS-CoV-2 mRNA in live cells, and pixel positivity was analyzed to determine viral replication as a function of viral mRNA.

**Figure 5:**
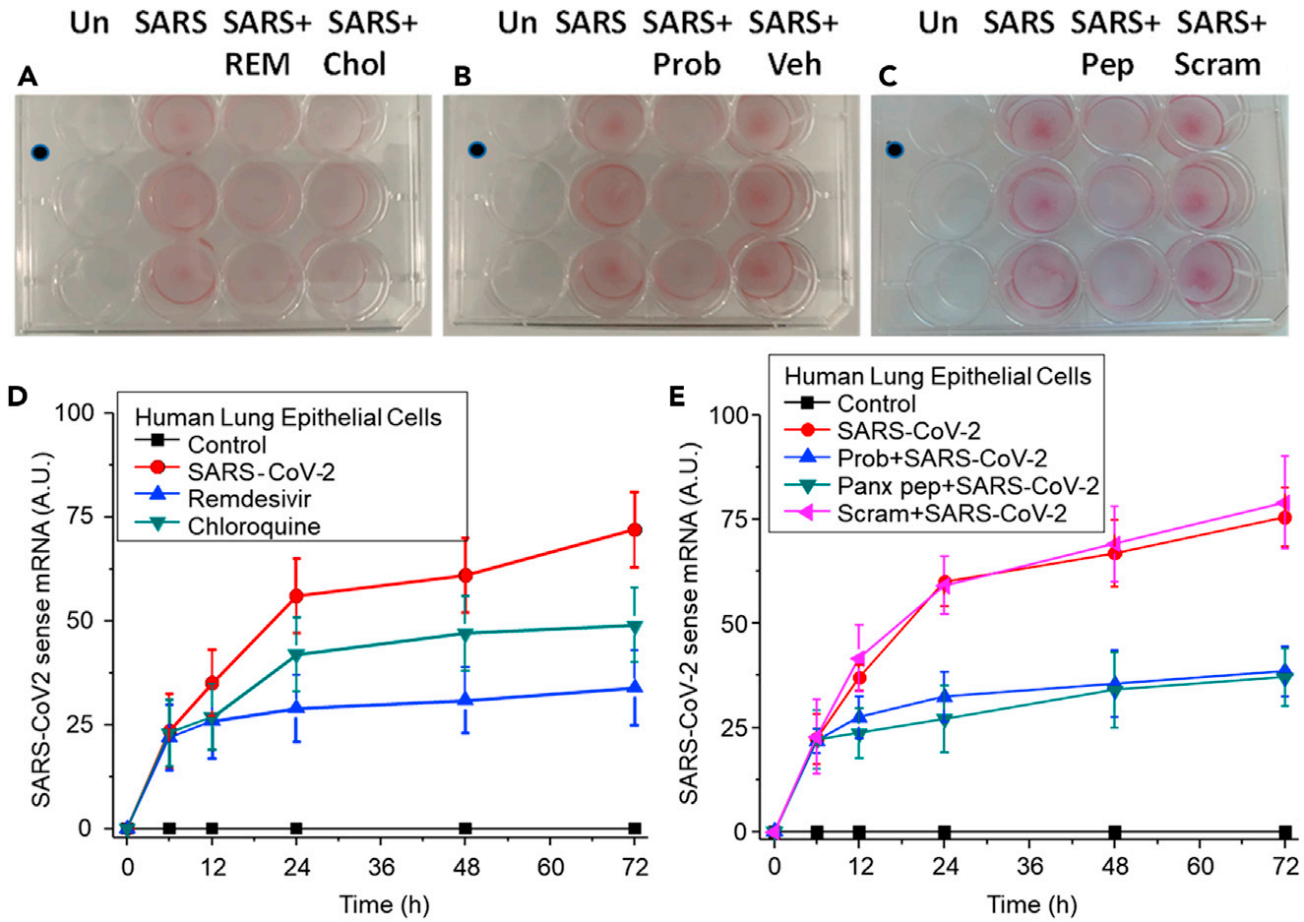
Viral entry and replication of SARS-CoV-2 infection in human lung epithelial cells Immunofluorescent staining via *in situ* hybridization to SARS-CoV-2 mRNA using the RNA scope V-nCov2019 sense probe was performed after treatment to measure viral replication. (A) Human lung epithelial cells were untreated (Un) or infected with SARS-CoV-2 (SARS) for 6 h and later treated with the anti-viral drugs remdesivir (3.5 μg/mL, REM) or chloroquine (10 μg/mL, Chlo) for 12 (represented), 24, 48, and 72 h (n = 3). (B) Human lung epithelial cells were untreated (Un) or infected with SARS-CoV-2 (SARS) for 6 h and later treated with the Panx-1 blocker, Probenecid (500 μM, REM), or vehicle (Veh) for 12 (represented), 24, 48, and 72 h (n = 3). (C) Human lung epithelial cells were untreated (Un) or infected with SARS-CoV-2 (SARS) for 6 h and later treated with the Panx-1 peptide (200 μM, Pep) or the Panx-1 scrambled peptide (200 μM, Scram) for 12 (represented), 24, 48, and 72 h (n = 3). (D) Fluorescence quantification of SARS-CoV-2 mRNA staining between 0 and 72 h for human lung epithelial cells treated with the SARS-CoV-2 virus (red circle), then remdesivir (blue upward triangle) or chloroquine (green downward triangle). Treatment with remdesivir significantly reduced viral replication at all time points sampled relative to infection with SARS-CoV-2 alone (p = 0.0013, n = 3). Treatment with chloroquine was not significant in reducing viral replication at all time points sampled relative to infection with SARS-CoV-2 alone (p ≤ 0.0743, n = 3). (E) Fluorescence quantification of SARS-CoV-2 mRNA staining between 0 and 72 h for human lung epithelial cells treated with the SARS-CoV-2 (red circle), then Probenecid (blue upward triangle), Panx-1 peptide (green downward triangle), or Panx-1 scrambled peptide (pink leftward triangle). Treatment with Prob or pep reduced viral replication at all time points sampled relative to infection with SARS-CoV-2 alone (p = 0.001, n = 3). Treatment with Scram was not significant in reducing viral replication at all time points sampled relative to infection with SARS-CoV-2 alone (p = 0.218, n = 3).

SARS-CoV-2 infection increased the mRNA detected by the sense probe in a time-dependent manner (Figure 5A, 24 h shown, and quantification in Figure 5D, SARS-CoV-2). Uninfected control plates did not show any nonspecific staining despite similar confluency as evaluated by Violet crystal staining. The addition of remdesivir (REM) at 6 h postinfection prevented the subsequent SARS-CoV-2 replication (Figure 5A and quantification in Figure 5D, blue, remdesivir). Values are significant compared with SARS-CoV-2 infection alone from 24 to 72 h (Figure 5A and quantification in Figure 5D, red, SARS-CoV-2, p = 0.0013, n = 3). Chloroquine (Chlo) treatment did not prevent viral replication at any of the tested times and was not significant compared with SARS-CoV-2 infection alone (Figure 5A and quantification in Figure 5D, green, Chloroquine, p ≤ 0.0743, n = 3). In contrast, Probenecid (Prob) and the Panx-1 mimetic blocking peptide (pep) prevented SARS-CoV-2 replication at all the tested times (Figures 5B and 5C and quantification in Figure 5E, blue and green, Prob + SARS-CoV-2 and Panx pep + SARS-CoV-2, respectively. All points were significant compared with SARS-CoV-2 infection alone from 12 to 72 h, p = 0.001, n = 3). The addition of vehicle (Veh) or the scrambled peptide (Scram) did not affect the time course of SARS-CoV-2 infection (Figures 5B and 5C, SARS + Veh and SARS + Scram, respectively). Overall, our data indicate that blocking the Panx-1 channel opening induced by SARS-CoV-2 could be used as an additional treatment to prevent replication and associated pathogenesis.

### Single-cell RNA sequencing from nasal epithelia obtained from COVID-19 individuals demonstrate a higher expression of Panx-1 mRNA

COVID-19 infection results in respiratory distress and symptoms such as coughing, congestion, and loss of taste or smell. These symptoms extend from the nasal cavity to the lungs and affect epithelial cells involved in respiratory function, such as immune and various luminal cells (Ahn et al., 2021; Mason, 2020). As described in the STAR Methods section, we used publicly available COVID-19 databases with the authorization of each group to reanalyze the data for Panx-1 mRNA expression in different cell populations of nasal epithelia collected from nasal swabs (Ballestar et al., 2020; Vieira Braga et al., 2019; Ziegler et al., 2021).

**Figure 6:**
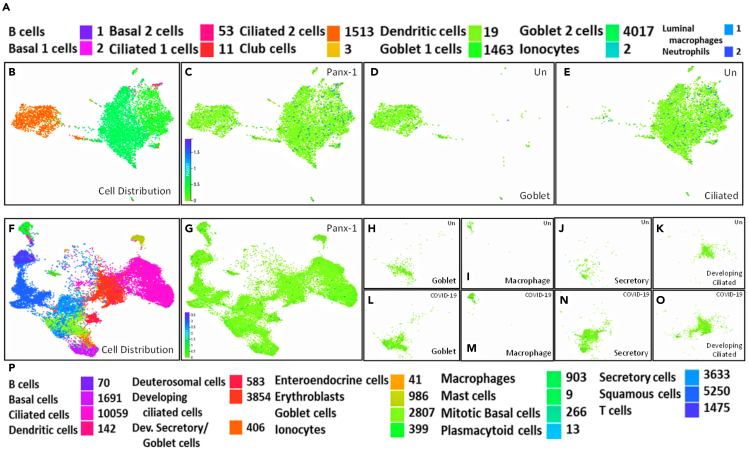
Single-cell expression of Panx-1 in nasal epithelial cells To determine the distribution and expression of Panx-1 in cells within control, uninfected, and COVID-19 patients, single-cell sequencing results were retrieved from the COVID-19 cell atlas online database for nasal epithelial cells and subcategories (https://www.covid19cellatlas.org/). The upper half of the figure represents cells collected and sequenced from one cohort of uninfected patients collected by the Vieira Braga lab. The lower half of the figure is representative of cells collected and sequenced from a second cohort consisting of both uninfected and COVID-19-infected patients collected by the Shalek lab. (A) The color scheme of cell-type distribution and cell count for uninfected patients expressing Panx-1. (B) Cell type distribution from 7,087 total cells. (C) Single-cell mRNA expression of Panx-1 in all distributed cell types. (D) Expression of Panx-1 in uninfected (Un) goblet cells. (E) Expression of Panx-1 in uninfected ciliated cells. (F) Cell-type distribution from 32,588 cells. (G) Single-cell mRNA expression of Panx-1 in all distributed cell types. (H) Expression of Panx-1 in uninfected (Un) goblet cells. (I) Expression of Panx-1 in uninfected macrophage. (J) Expression of Panx-1 in uninfected secretory cells. (K) Expression of Panx-1 in uninfected developing ciliated cells. (L) Expression of Panx-1 in COVID-19 goblet cells. (M) Expression of Panx-1 in COVID-19 macrophage. (N) Expression of Panx-1 in COVID-19 secretory cells. (O) Expression of Panx-1 in COVID-19 developing ciliated cells. (P) The color scheme of cell-type distribution and cell count for uninfected and infected patients expressing Panx-1.

Using 10X Genomics Chromium droplet single-cell RNA sequencing, epithelial cells were profiled by the Vieira Braga et al. (2019) and Shalek groups to generate airway nasal epithelial datasets (Ballestar et al., 2020). The green to blue color heat scaling represents the expression level of Panx-1 at the single-cell resolution, and the cells are grouped based on the cell types specified. The Vieira dataset (Figures 6A–6E) was collected from an upper airway nasal brush from uninfected patients. A map corresponding to the different identities of cell populations (Figure 6B) is denoted for cell type and quantity (Figure 6A). The total expression of Panx-1 mRNA for the entire population detected is also shown (Figure 6C). Representative goblet and ciliated cells corresponded to the more abundant cell types with the highest relative levels of Pax-1 mRNA (Figures 6D and 6E). Overall, Panx-1 mRNA expression is expressed at low levels in most cells.

In contrast, using the Shalek et al. (Ballestar et al., 2020) dataset, in COVID-19 conditions, the quantity and cell types in the nasal epithelia increased (Figure 6F represents cell distribution, and Figure 6P corresponds to the quantity and cell types identified). Analysis of the cell population expressing Panx-1 mRNA indicates that most cells in the nasal epithelia from COVID-19 patients were positive (Figure 6G). Analysis of goblet, macrophages, secretory, and developing ciliated cells in uninfected conditions (Figures 6H–6K, respectively) compared with COVID-19 samples (Figures 6L–6O) indicate that these populations were expanded and the expression of Panx-1 mRNA also increased. Nearly the entire population of cells in the nasal epithelia swabs becomes positive for Panx-1 mRNA, supporting our hypothesis that Panx-1 plays a key role in the SARS-CoV-2 pathogenesis.

### Analysis of lung tissues obtained from lethal COVID-19 cases shows a high level of Panx-1 protein expression

We next conducted immunofluorescence staining for Panx-1 and other cellular markers to determine the expression and distribution in human lung tissue samples using confocal microscopy under control and COVID-19 conditions. Tissue samples infected with SARS-CoV-2 were obtained from a rapid autopsy process from Assistance Publique-Hopitaux de Paris (AP-HP) at Raymond Poincaré Hospital, Garches, France and UTMB. As a control, uninfected lung biopsy and tumor border tissues (tumor healthy tissue) were obtained from the Anatomic Pathology Laboratory at UTMB. To ensure an unbiased assessment, patient personal information was not collected during data collection, and all samples were received and analyzed blindly. After all the data were acquired, clinical and COVID-19 status was requested to guarantee proper scientific rigor.

**Figure 7:**
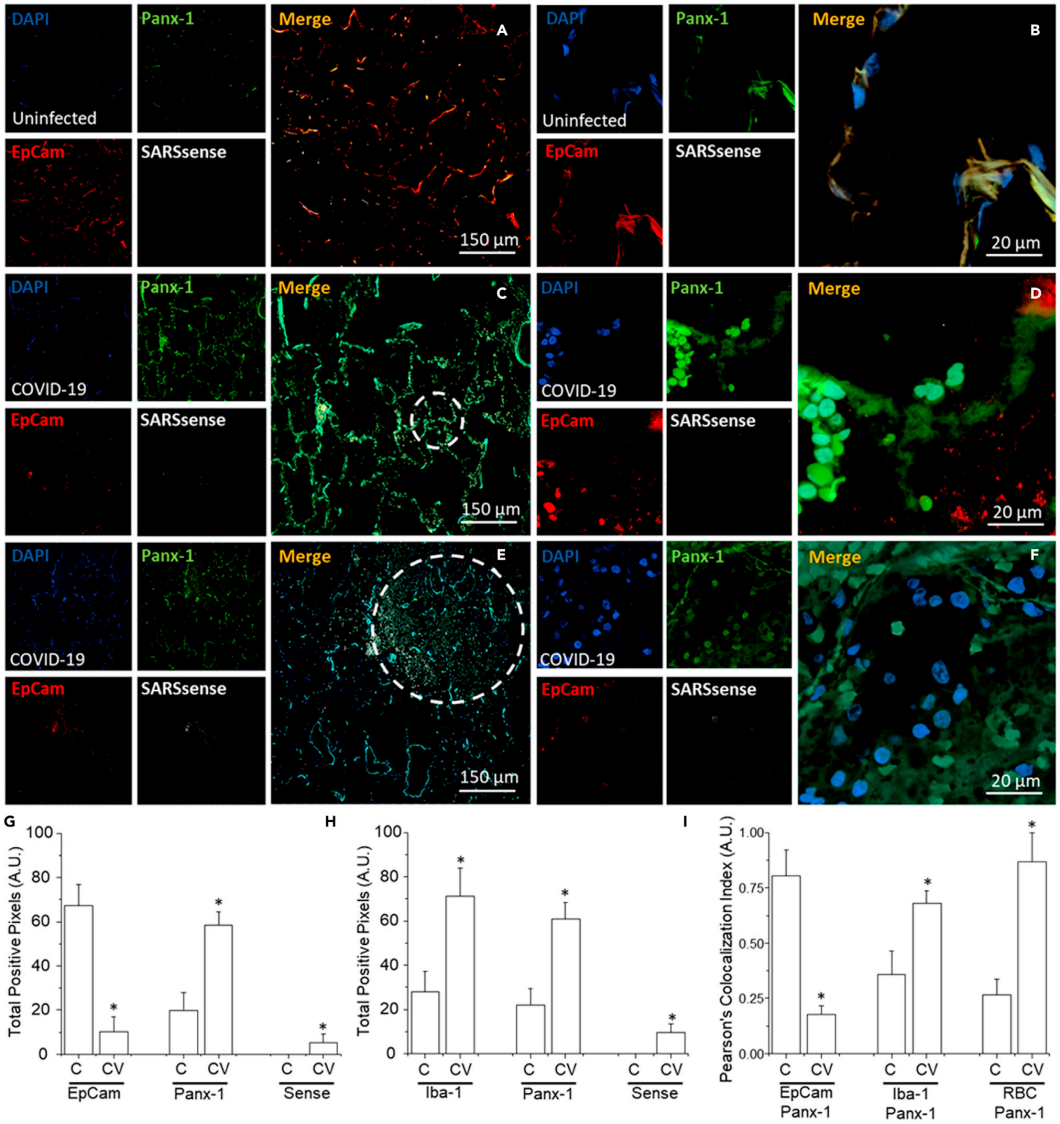
Panx-1 channel opening is essential for SARS-CoV-2 replication Immunofluorescence staining was performed on re-sectioned human lung tissue biopsied from patients with COVID-19 or tumors biopsied from patients with lung cancer as a control. Confocal microscopy was conducted to determine the expression and distribution of Panx-1 and other cellular markers. Tissues were stained for DAPI (a nuclear marker, blue), Panx-1 (green), EpCam (an epithelial cell marker, red) or Iba-1 (a macrophage marker, not shown), and SARSsense (a probe for SARS-CoV-2 mRNA using the RNAscope V-nCoV2019-sense probe). (A) Staining of uninfected tumor lung tissue for DAPI, Panx-1, EpCam, and SARSsense at 10× magnification and (B) 40× magnification (n = 3). No significant levels of SARSsense staining for viral mRNA were detected. Note that EpCam and Panx-1 are expressed at the borders of an intact alveolar wall. (C) Staining of lung tissue from patients infected with SARS-CoV-2 for DAPI, Panx-1, EpCam, and SARSsense at 10× magnification and (D) 40× magnification (n = 6). Low but significant levels of SARSsense staining for viral mRNA were detected. Significant levels of EpCam and Panx-1 expression were detected, with expressions elevated near the alveolar wall. Note that for this sample, the morphological integrity of the alveolar wall is retained. (E) Staining of lung tissue from patients infected with SARS-CoV-2 for DAPI, Panx-1, EpCam, and SARSsense at 10× magnification and (F) 40× magnification (n = 5). Significant levels of SARSsense staining for viral mRNA were detected. Decreased but significant levels of EpCam and Panx-1 were detected. Note that for this sample, the morphological integrity of the alveolar wall has been compromised (represented by the dashed circle). (G) Quantification of the total pixels per area for the control (C) and COVID-19 (CV)-infected conditions was low but significant for EpCam (CV, p = 0.00025, n = 9 cases with 6 sections per individual). In contrast, Panx-1 expression was high and significant (CV, p = 0.00153, n = 9 cases with 6 sections per individual). SARSsense (Sense) staining for mRNA was low but significant. (H) Quantification of the total pixels per area for the control (C) and COVID-19 (CV)-infected conditions for Iba-1 was high and significant (p = 0.0031, n = 9, not shown). Similarly, Panx-1 expression was high and significant (p = 0.00102, n = 9). SARSsense (Sense) staining for mRNA was again low but significant. (I) Quantification of the Pearson's colocalization index between EpCam, Iba-1, and RBC with Panx-1 in the control (C) and COVID-19 (CV)-infected conditions. EpCam with Panx-1 staining in COVID-19-infected patients was decreased relative to the control and indicated progressive deterioration of the alveolar wall (p = 0.001, n = 9). In contrast, Iba-1 with Panx-1 staining in COVID-19-infected patients was ∼2-fold higher relative to the control and indicated an increase in expression of Panx-1 in macrophages (p = 0.001, n = 9). Similarly, red blood cell (RBC) hemoglobin autofluorescence to Panx-1 staining indicates that the expression of Panx-1 increased in red blood cells of COVID-19-infected patients (p = 0.0025, n = 9).

Staining was conducted for DAPI (a nuclear marker), Panx-1, EpCam (an epithelial marker) or Iba-1 (a macrophage marker), and SARSsense (a probe for SARS-CoV-2 mRNA using the RNAscope V-nCoV2019 sense probe). Analysis of control uninfected tissues indicates that Panx-1 protein was expressed in epithelial cells and macrophages at the alveolar wall (Figure 7A; a higher magnification of the alveolar wall is shown in Figure 7B). As predicted, no staining for the SARSsense probe was detected in the controls (Figures 7A and 7B, SARSsense). In contrast, Panx-1 expression in COVID-19 cases (n = 9 different individuals with at least 8–12 days in an intensive care unit) exhibits a significant increase in the numbers of cells expressing Panx-1 expression detected in the alveolar wall compared with uninfected lungs (Figures 7C and 7D). In addition, a significant loss of EpCam was detected in all patient samples analyzed, denoting tissue compromise (Figures 7C–7F).

COVID-19 infection was confirmed by the detectable expression of SARSsense. Analysis of lungs obtained from COVID-19 patients with severe immune-cell-infiltrating pathology and major alveolar wall compromise indicates a significant upregulation of Panx-1 expression in multiple cell types, as predicted from the scRNAseq data. These cells exhibit a higher degree of damage as indicated by a less defined but still increased localization of Panx-1 (Figures 7C–7F, see dotted circle).

Quantification of the total pixels per area (100 A.U. representing saturation, A.U.) indicates that staining for EpCam was high for the control lung tissue in control conditions. All COVID-19 cases analyzed indicated that EpCam staining was at low to undetectable levels, demonstrating the large degree of lung damage (Figure 7G, Control [C] versus COVID-19 [CV] EpCam, p = 0.00025, n = 9 cases with 6 sections per individual). Analysis of Panx-1 protein expression indicates that under control conditions (Figure 7G, C-Panx-1), Panx-1 expression was moderate, but under COVID-19 conditions, Panx-1 expression increased significantly (Figure 7G, CV, Panx-1, p = 0.00153, n = 9 cases with 6 sections per individual). The fluorescence emission for the SARS-CoV-2 sense probe, SARSsense, was low but significant enough to conclude the presence of the viral RNA in COVID-19 conditions (Figure 7G, CV, Sense). Quantification of macrophages, which are positive for Iba-1, indicates that resident macrophages were abundant at the alveolar wall under control conditions (Figure 7H, C, Iba-1). However, in SARS-CoV-2-infected lungs, infiltration of macrophages was significantly higher (Figure 7H, CV, Iba-1, p = 0.0031, n = 9 cases with 9 sections per individual) in the alveolar wall associated with immune cell infiltration and lung parenchyma destruction (Figure 7E, dotted circle area). The analysis of Panx-1 and SARS-CoV-2 mRNA expression and distribution in tissue stained in combination with Iba-1, then, was similar to the data for tissue stained for Panx-1 and SARS-CoV-2 mRNA in combination with EpCam (Figure 7H compared with 7G, respectively, p = 0.00102, n = 9 cases with 9 sections per individual). Further examination of the colocalization between the cellular markers EpCam, Iba-1, and RBC (by hemoglobin autofluorescence) indicates that macrophages had a high Panx-1 protein expression (Figure 7I, Iba-1 to Panx-1, p = 0.001) and RBC (Figure 7I, RBC to Panx-1, p = 0.0025, n = 9 cases with six sections per individual) in COVID-19 patients analyzed by the Pearson's colocalization index. In contrast, Panx-1 expression was increased in the remaining cells that were positive for EpCam (Figure 7I, EpCam to Panx-1, p = 0.001, n = 9 cases with 6 sections per individual). This experiment indicates that Panx-1 expression is increased in COVID-19-infected tissues and is widely distributed into multiple cell types, especially in areas with compromised immunity and tissue destruction.

### Analysis of bronchiolar alveolar lavage from COVID-19 individuals confirms the accumulation of ATP, PGE_2_, and IL1β

In healthy individuals, the BAL contains low populations of macrophages (80%–90%), lymphocytes (5%–10%), neutrophils (∼3%), and eosinophils (≤1%), and any changes in these proportions are an indication of disease (Meyer, 2007; Meyer and Zimmerman, 2002). scRNAseq identified several cell types from the BAL, including immune cells (Berman et al., 2021; Stanczak et al., 2021) and lung cells (Berman et al., 2021), supporting the concept of extensive lung damage within COVID-19 cases, as indicated in Figure 6. Most of these cells are released into the nasal scrape, and lung staining indicates a widespread expression of Panx-1 mRNA and protein that, *in vitro*, correlates with the secretion of ATP, PGE_2_, and IL-1β release.

**Figure 8:**
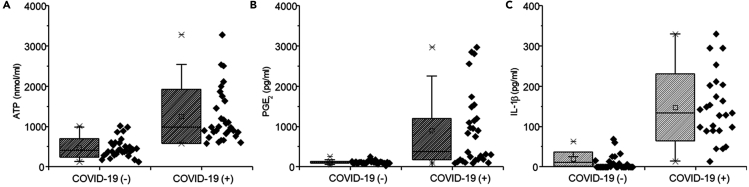
BAL obtained from non-COVID-19 and COVID-19 individuals denote a high concentration of inflammatory mediators released by opening Panx-1 channels BAL was obtained from individuals with COPD, n = 30 for the non-COVID-19 conditions (COVID-19(−)) or with mild/acute COVID-19 (COVID-19 (+)), n = 30. BAL was inactivated using temperature (65°C for 30 min), and ELISA for (A) ATP, (B) PGE_2_, and (C) IL1β was performed according to the manufactured instructions. BAL from COVID-19 individuals had higher amounts of these mediators as compared with non-COVID-19 individuals. ATP, p = 0.0064 compared with COVID-19(−); PGE_2_, p = 0.0014 compared with COVID-19(−) and IL-1β, p = 0.0031 compared with COVID-19(−), n = 30.

Thus, to determine, *in vivo,* whether these products are concentrated in patient lung secretions, we quantified these inflammatory factors in BAL by ELISA. The BAL was collected from patients living with COPD for at least 8 years (10.02 ± 5.04 years, n = 30 different individuals). Our findings indicate that in control uninfected patients, the levels of secreted ATP (Figure 8A, COVID-19(−)), PGE_2_ (Figure 8B, COVID-19(−)), and IL-1β (Figure 8C, COVID-19(−)) are significant (Figures 8A–8C, respectively, p = 0.00161, n = 30). Higher concentrations of ATP (Figure 8A, COVID-19(+), p = 0.0064, n = 30), PGE_2_ (Figure 8B, COVID-19(+), p = 0.0014, n = 30), and IL-1β (Figure 8C, COVID-19(+), p = 0.0031, n = 30) were detected in the BAL from COVID-19 individuals who survived infection. This experiment, therefore, demonstrates that intracellular inflammatory factors such as ATP, PGE_2_, and IL-1β are released through the opening of Panx-1 channels and are highly concentrated in the BAL.

## Discussion

COVID-19 is an unprecedented pandemic that mainly affects the respiratory and immune systems. The rapid development and administration of vaccines has stymied its uncontrolled spread, resultant hospitalization, and death (Frederiksen et al., 2020; Hodgson et al., 2021; Ledford, 2020; Zhao et al., 2020). However, the emergence of new SARS-CoV-2 variants and the long-term consequences of the infection are still unknown. Therefore, understanding the pathogenesis of SARS-CoV-2 and progression into COVID-19 is urgent.

Here, we identified that Panx-1 channels become open upon binding the SARS-CoV-2 spike protein, the hCoV-229E virus, or its S protein. The Panx-1 opening upon SARS-CoV-2 infection was dependent upon ACE-2, furin, and endocytosis and that the Panx-1 opening resulted in the release of the pro-inflammatory biomolecules such as ATP, PGE_2_, and IL-1β into the extracellular space. Analysis of Panx-1 expression and distribution indicates that SARS-CoV-2 infection and COVID-19 disease are associated with enhanced inflammation and suggests that targeting Panx-1 channel opening or the subsequent release of intracellular inflammatory factors could provide alternative mechanisms of preventing COVID-19-associated damage or mitigating disease progression. Therefore, we propose that Panx-1 channels are a new host protein pathway required for SARS-CoV-2 infection and signaling.

Panx-1 channels participate in several inflammatory conditions exacerbated during COVID-19 pathogenesis, including hypoxia, coagulation, blood pressure, endothelial permeability, and apoptosis (Abdeen et al., 2021; AbdelMassih et al., 2021; Chang et al., 2020; Contoli et al., 2021; Hertzog et al., 2021; Li et al., 2020; Liu et al., 2020b; Montero et al., 2020; Page et al., 2021; Taz et al., 2021; Toldo et al., 2021; Wang et al., 2020). We propose that upon Panx-1 channel opening, several biomolecules are released into the extracellular space and result in modulation of COVID-19 pathogenesis: first, ATP release and modification of local signaling enable viral entry through the activation of purinergic receptors; second, IL-1β release results in a pro-inflammatory response and recruitment of leukocytes into the area of infection (Kim et al., 2015); and third, the release of PGE_2_ into the extracellular matrix and its role in coagulation/vascular compromise (Friedman et al., 2015; Gross et al., 2007) highlight its importance in extensively vascularized regions that become damaged when challenged by conditions such as SARS-CoV-2 infection. These three mechanisms and their dysregulation during COVID-19, therefore, may partially or cumulatively contribute to the pathogenesis and progression of SARS-CoV-2 infection.

In HIV, we previously determined that the binding of gp120 to CD4 and CCR5/CXCR4 receptors and co-receptors induces opening of Panx-1 channels, ATP release, and subsequent purinergic receptor activation to enable HIV entry and subsequent undefined replication steps in macrophages and T cells (Gajardo-Gomez et al., 2020; Hazleton et al., 2012; Orellana et al., 2013; Velasquez and Eugenin, 2014). We believe that this specific gp120 trigger results in intracellular calcium signaling and actin rearrangement that allows HIV to fuse with the host plasma membrane (Gajardo-Gomez et al., 2020; Hazleton et al., 2012; Velasquez et al., 2020). SARS-CoV-2 infectivity is initiated by binding of the virus to ACE-2, mainly expressed in the lung, kidney, and vascular endothelium (Zamorano Cuervo and Grandvaux, 2020; Zou et al., 2020). A critical difference between SARS-CoV-2 and other coronaviruses is the furin (or protease)-dependent site that results in a 10-fold increase in the binding of ACE-2 to promote viral entry (Walls et al., 2020a). This site is at the S1/S2 boundary of SARS-CoV-2 S protein (Walls et al., 2020c). This difference has been associated with enhanced infectivity and potentially contributes to the high pathogenesis of SARS-CoV-2 over other coronaviruses.

Interestingly, this type of mutation or adaptation has been observed in several highly pathogenic viruses such as avian influenza and Newcastle virus (Klenk and Garten, 1994; Steinhauer, 1999; Walls et al., 2020c). These changes could increase viral infectivity, transmissibility, and pathogenesis of viral infection. Subsequently, when we compared the effects of SARS-CoV-2 infection with hCoV-229E viral infection, Panx-1 opening and its sensitivity to furin, ACE-2, and endocytosis were more evident with SARS-CoV-2, suggesting that the S protein of SARS-CoV-2 is not only important for entry but also associated with inflammation and the pathogenesis of the virus. In addition, our ATP and PGE_2_ data demonstrated that the SARS-CoV-2 S protein, in contrast to the hCoV-229E virus, induced the release of these inflammatory factors. The differences in inflammation and Panx-1 channel opening between SARS-CoV-2 and hCoV-229E viruses cannot be explained due to MOI or protein concentrations due to the fact that IL1β secretion was similar. In the future, it will be interesting to determine whether the recent vaccines for COVID-19 can prevent viral entry and Panx-1-associated inflammation or whether both effects could be separated.

Normally, Panx-1 channels exist in a closed state in healthy conditions (Pelegrin and Surprenant, 2006; Saez et al., 2010; Seror et al., 2011; Swayne and Boyce, 2017). Thus, targeting Panx-1 opening could have minimal side effects in healthy individuals and prevent viral infection and spread generated by multiple SARS-CoV-2 variants. For example, Probenecid is an FDA-approved drug to treat gout and is an excellent Panx-1 blocker, as demonstrated in our previous studies. Probenecid prevented several HIV entry/replication steps (Anthonypillai et al., 2004; Dahl and Keane, 2012; Jorajuria et al., 2004; Liu et al., 2020a; Lucia et al., 2005; Thomas, 2004), influenza A (Chairat et al., 2013; Holodniy et al., 2008; Howton, 2006; Marquez-Dominguez et al., 2020; Rosli et al., 2019; Wattanagoon et al., 2009), and gout (Drugs for gout, 2019; Chuang et al., 2020; Rubinstein et al., 2020; Wei et al., 2015) but also prevented general inflammation. Several groups have proposed Probenecid and other Panx-1 blockers to have at least three different modes of action: first, direct participation in infection and replication; second, prevention of the inflammasome activation; and lastly, maintenance of effective concentrations of drugs inside of the cells. This last one is of particular interest because when the Panx-1 channel opens to release ATP and other intracellular factors, drugs that need to reach an effective intracellular concentration can “leak” into the extracellular space through these same channels. In addition, Panx-1 blockers also reduce the secretion of proteases into the extracellular space, such as cathepsins (Swayne et al., 2020). Extracellular proteases, such as furin and cathepsins, promote SARS-CoV-2 infection over other coronaviruses, as demonstrated by others (Bestle et al., 2020; Dessie and Malik, 2021; Huffman et al., 2021; Murgolo et al., 2021; Raghav et al., 2021; Rossi et al., 2020; Watzky et al., 2021; Zhang et al., 2021), and our current data support these findings by identifying a novel mechanism of virus-mediated inflammation. Our data that ATP, PGE_2_, and IL1β are highly concentrated in the BAL obtained from COVID-19 individuals is exciting but needs to be considered carefully due to the fact that during the evolution of the disease, significant apoptosis of lung cells occurs (Liu et al., 2021). This apoptosis then contributes to the nonspecific release of ATP.

Glycyrrhizic acid derivatives such as 18-α/β glycyrrhetinic acid and carbenoxolone are most likely the most common gap junction and Panx-1 channel blockers (Patel et al., 2014). Glycyrrhizic acid inhibits SARS-CoV replication in Vero cells with high selectivity while conferring high protection with minimal adverse reactions (Hoever et al., 2005; Ming and Yin, 2013; Utsunomiya et al., 1997). These compounds are currently under clinical trial examination (https://clinicaltrials.gov/ct2/show/NCT04487964) as a complementary intervention for COVID-19 treatment, despite having currently unknown mechanisms of action. Our data provide an explanation by which glycyrrhizic acid derivatives can prevent and protect from the damaging effects of SARS-CoV-2. Only recently, in the context of HIV/SIV infection, have we determined that the Panx-1 mimetic peptide is not toxic to primates, retains stability in circulation in the body, blocks Panx-1 effectively, and prevents synaptic compromise induced by SIV (Gorska et al., 2021), suggesting that even the mimetic peptide can be used as an alternative treatment as a Panx-1 channel blocker to prevent further SARS-CoV-2 infection and its associated inflammation.

ATP and its metabolites have become a critical component of multiple diseases, including HIV and COVID-19 (Abouelkhair, 2020; Alves et al., 2020; de Oliveira et al., 2021; Dos Anjos et al., 2020; Edwards, 2021; Fonteles et al., 2020; Franciosi et al., 2021; Klaver and Thurnher, 2021; Mishra et al., 2021; Pacheco and Faria, 2021; Shan et al., 2020; Simoes and Bagatini, 2021). Our data in long-term HIV-infected individuals indicate that several chronic effects of HIV are correlated with ATP dysfunction and vascular disease, such as cognitive impairment (Velasquez et al., 2020). A critical characteristic of the COVID-19 tissues analyzed is the localized infiltration of immune cells, fibrosis, hemorrhagic events, and intravascular coagulation, unlike other diseases. Our data indicate that platelets are a key component of the SARS-CoV-2 amplification cycle by providing an alternative source of the virus. Furthermore, detection of megakaryocytes in the BAL correlate with worsened disease outcome. This background and data from this current paper indicate an intersection among coagulation, ATP, and pathogenesis. Infiltration of the SARS-CoV-2 into the lungs results in ATP release through Panx-1 channels and leads to several dysfunctions of vasculature and signaling and potentially cognitive issues as in the case of HIV. Interestingly, ATP release through Panx-1 channels may have more far-reaching implications than just as a marker for viral infection or future disorders. ATP dysregulation potentially also implies the exacerbated dysregulation of other pathways that result in a cycle of chronic symptoms resulting from viral infection.

Human platelets have purinergic receptors that are further delineated into categories based on adenosine, ADP- or ATP-bound ligand, which may function as neurotransmitters and signaling molecules. These different purinergic receptors fall under G-protein-coupled receptors (GPCRs), including P2Y_12_, P2Y_1_, P2X_1_, and other ligand-gated ion channels. Extracellular ATP can also activate these receptors to increase membrane permeability due to the opening of Panx-1 channels, as we demonstrated in this paper. The subsequent signaling of adenosine-based molecules with purinergic receptors may result in feedback where the receptors participate in further replication and viral entry (Abbracchio and Ceruti, 2006; Hechler and Gachet, 2015). Upon activation, platelets become prothrombotic and accelerate the release of pro-inflammatory factors to promote vasoconstriction and coagulation (Gachet and Hechler, 2020; Grenegard et al., 2008; Mahaut-Smith et al., 2016; Manica et al., 2018; Oury and Wera, 2021). It is also known that purinergic and thrombin can synergize (Dorsam and Kunapuli, 2004; Nylander et al., 2003, 2004) to contribute to the extensive coagulation observed during COVID-19 pathogenesis. Platelet activation by ATP and thrombin further promote ADP release from dense granules (Woulfe et al., 2001), potentially resulting in a vicious cycle observed in deadly cases of COVID-19. Other purinergic receptors, such as P2Y_2_, P2Y_6_, and P2X_7_, could also contribute to pathological effects in pulmonary cell types. P2Y_2_, activated by ATP but not ADP (Abbracchio and Ceruti, 2006), regulates endothelial inflammation, most notably promoting the adhesion and chemotaxis of inflammatory cells (Alberto et al., 2016; Burnstock, 2017; Buscher et al., 2006; Gabl et al., 2015; Liu et al., 2016; Marcet et al., 2004; Muhleder et al., 2020; Sophocleous et al., 2020; van Heusden et al., 2020). P2Y_12_ has also been demonstrated to contribute to endothelial cell pathology (Sidiropoulou et al., 2021; Vemulapalli et al., 2020). Purinergic receptor activation has also been implicated in the dysregulation of Ca^2+^ signaling and wave propagation (MacVicar and Thompson, 2010). Interestingly, other studies have also demonstrated that Ca^2+^ signaling may result from direct calcium leakage through Panx-1-mediated calcium channels out to the extracellular space and affect intercellular communication, wave propagation, and homeostasis (Vanden Abeele et al., 2006). Panx-1 channel permeability and opening potentially have far-reaching implications in several pathways that we have partially addressed in this manuscript; however, we still need to examine the distribution, function, and potential therapeutic use of these receptors to prevent or revert the early and long-term consequences of SARS-CoV-2 infection.

### Limitations of our study

First, the mechanism of Panx-1 channel opening and ATP secretion induced by SARS-CoV-2 S protein, but not by the hCoV-229E virus, is unknown. However, it is dependent on furin activity, but how this cleavage alters channel gating and permeability is unknown. Second, future studies will determine the mechanisms of Panx-1 upregulation in the lung. Lastly, the purinergic receptor involvement in resident and infiltrated cells and vascular dysfunction need to be determined to understand the time course of infection, inflammation, damage, and recovery or death, as well as the long-term consequences of the disease. Additional research in these areas could illuminate specific treatment windows to prevent the devastating acute and chronic consequences of COVID-19 as well as potential emerging variants.

## STAR★Methods

### Key resources table

**Table:** 

REAGENT or RESOURCE	SOURCE	IDENTIFIER
**Antibodies**		

Panx-1	Thermofisher	Cat#487900, RRID: AB_2532253
Iba-1	Abcam	Cat#ab5076, RRID: AB_2224402
EpCAM	Abcam	Cat#ab7504, RRID: AB_305949
Donkey Anti-Rabbit Alexa 488	Thermo Fisher	Cat#A21206, RRID:AB_2535792
Donkey Anti-Goat Alexa 568	Thermo Fisher	Cat#A11057, RRID:AB_2534104
Goat Anti-Mouse Alexa 568	Thermo Fisher	Cat#A11031, RRID: AB_144696

**Biological samples**		

Primary Small Airway Epithelial Cells; Normal, Human	ATCC	Cat#PCS-301-010

**Cell lines**		

Airway Epithelial Cell Basal Medium	ATCC	Cat#PCS-300-030

**Chemicals**		

DMEM	ThermoFisher Scientific	Cat# 11995-065
Bronchial Epithelial Growth Kit	ATCC	Cat#PCS-300-040
Dulbecco’s Phosphate-buffered saline	gibco	Cat#14190-144
Dako Pen	Dako	Cat# S2002
0.05% Trypsin EDTA	ThermoFisher Scientific	Cat# 25300-054
Penicillin/Streptomycin	ThermoFisher Scientific	Cat#15140-122
Fetal bovine serum	ThermoFisher Scientific	Cat# 16000044
Paraformaldehyde	Electron Microscopy Sciences	Cat# 15710-S
Fish gelatin	Sigma-Aldrich	Cat# G7041
BSA (Bovine serum albumin)	Sigma-Aldrich	Cat# 05470
Horse serum	Sigma-Aldrich	Cat# H0146
EDTA	Invitrogen	Cat#15575-020
Xylene	Fisher Chemical	Cat#X3S-4
Ethanol 200proof	Decon Labs, Inc.	Cat#2701
Tris-buffered saline	Fisher-Bioreagents	BP2471-1
Triton x-100	Sigma	X100
Ethidium Bromide Solution	ThermoFisher Scientific	Cat#15585-011
ProLong™ Gold Antifade Mounting with DAPI	ThermoFisher Scientific	Cat# P36931
SigmaFast DAB Tablet	Sigma-Aldrich	Cat#D-4168

**Chemicals**		

RNAscope Target retrieval solution	ACD	Cat# 322000
RNAscope Wash Buffer	ACD	Cat# 310091
RNAscope Multiplex Fluorescent Detection Reagents V2	ACD	Cat# 323110
RNAscope LS Multiplex TSA Buffer	ACD	Cat# 322810
Probenecid	Sigma	Cat# P8761-25G
Panx-1 mimetic blocking peptide	Origene	Sequence: WRQAAFVDSY
Panx-1 scrambled peptide	Origene	Sequence: FADRYWAQVS
SARS-CoV-2 Spike Membrane protein	Origene	Cat# TP701119
hCoV-229E Spike Membrane protein	Origene	Cat# VC100563

**Probes**		

SARS-CoV-2 (Sense) probe	ACD	Cat# 845701
SARS-CoV-2 (S) probe	ACD	Cat# 848561

**Software**		

NIS-Elements-AR	Nikon	https://www.microscope.healthcare.nikon.com
Nano Zoomer Digital Pathology	Hamamatsu	https://www.hamamatsu.com/us/en/product/type/U12388-01/index.html

### Resource availability

#### Lead contact

• Further information and request for resources and reagents should be directed to and fulfilled upon reasonable request by the Lead contact Eliseo Eugenin (eleugeni@utmb.edu).

#### Materials availability

• This study did not generate new unique reagents.

### Experimental model and subject details

#### Primary cultures

##### Human primary airway epithelial cells

Human Primary Small Airway Epithelial Cells (ATCC Cat#: PCS-301-010) were purchased from ATCC (Manassas, VA) and derived from a white 34-year old male with no history of smoking (Lot: 70026720). Airway epithelial cells were cultured in 60x15 mm cell culture dishes (Greiner CELLSTAR Cat#: 628160), and Airway Epithelial Cell Basal Medium (ATCC Cat#: PCS-300-030) supplemented with Bronchial Epithelial Cell Growth Kit (ATCC Cat#: PCS-300-040) and penicillin/streptomycin at 37^c^ and 5% CO_2_.

### Method details

#### Materials

RPMI medium (Cat:11875-093), penicillin/streptomycin (Cat:15140-122), trypsin-EDTA (Cat:25300-054), and Ethidium (Etd) Bromide (Cat: 15585011) were purchased from Thermofisher (Carlsbad, CA). Fetal bovine serum (Cat: S11150H) was purchased from R&D Systems (Minneapolis, MN). The SARS-229E isolate was obtained from the ATCC (ATCC Cat: VR-740). SARS-CoV-2 Spike-Membrane protein was purchased from OriGene (Cat: TP701119). Recombinant ACE-2 and furin (R and D). Recombinant S protein from virus 229E (Creative Diagnostic, NY). All other reagents were purchased from Sigma-Aldrich (St. Louis, MO) unless otherwise designated.

#### Methods

##### SARS-CoV-2 and hCoV-229E-infection and S-protein treatment

SARS-CoV-2 (GISAID, EPI_ISL_406862) and Human Coronavirus 229E (ATCC, VR-740) were propagated in Vero E6 cells. SARS-CoV-2 experiments were conducted in a biosafety level 3 (BSL3) facility as indicated in the biosafety guidelines of the Center for Disease Control (CDC) and the National Institute of Health (NIH). Cells were treated with SARS-CoV-2 S and recombinant 229E S protein at 1 μg/mL for the time indicated in the result section (Origene, MD).

##### Blocking peptides and siRNA

Three unique 27mer siRNA duplexes against human Panx-1 were predesigned and obtained from Origene (Rockville, MD). Experiments were carried out 2 days post-transfection. Also, we used a siRNA to Cx43 was used as a control. The Panx-1 mimetic blocking peptide ^10^Panx-1 (WRQAAFVDSY) and the scrambled peptide (FADRYWAQVS) were synthesized by Peprotech, NJ.

##### Dye uptake and time-lapse fluorescence imaging

To characterize the functional state of Panx-1 channels, dye uptake experiments using ethidium (Etd) bromide were performed as described previously (Gajardo-Gomez et al., 2020; Gorska et al., 2021). Cells were washed twice in Hank's balanced salt solution and then exposed to Locke’s solution (containing: 154 mM NaCl, 5.4 mM KCl, 2.3 mM CaCl_2_, 5 mM HEPES, and pH 7.4) with 5 μM Etd, and time-lapse microscopy was performed. Phase-contrast and fluorescence microscopy with time-lapse imaging were used to record cell appearance and fluorescence intensity changes in each condition. Fluorescence was recorded every 30 sec up to 240 min. The NIH ImageJ program was used for offline image analysis and fluorescence quantification. For data representation and calculation of Etd uptake slopes, the average of two independent background fluorescence intensity measurements (FB, expressed as arbitrary units, AU) was subtracted from fluorescence intensity in each cell (F1). This calculation (F1-FB) showed that at least 21-42 cells were averaged and plotted against time (expressed in minutes). Slopes were calculated using Microsoft Excel software and expressed as A.U./min. Microscope and camera settings remained the same in all experiments. Dead cells or cells with a damaged plasma membrane were identified during the time-lapse microscopy due to their non-specific Etd uptake determined by lack of time dependency. Dead cells were not quantified.

##### Electrophysiology

Cells were plated on glass coverslips 1-2 d before recordings. Whole-cell patch-clamp recordings were performed on cells bathed in HEPES supplemented phosphate-buffered saline (H-PBS) containing (mM): NaCl 147, HEPES 10, CaCl_2_ 2, MgCl_2_ 1 and KCl 2.7, Na_2_HPO_4_ 8, and KH_2_PO_4_ 2, pH 7.4. The pipette solution contained (mM): CsCl 130, EGTA 10, HEPES 10, CaCl_2_ 0.5, pH 7.4. Opening of Panx-1 channels by voltage was performed by applying 1 sec -10 mV pulse, followed by 12 s voltage ramps from a holding potential of −70mV to +70mV followed by 1 s steps to 0 and -30 mV to evaluate possible tail currents. To analyze Panx-1 channels, epithelial cells were exposed to either control (C) or test (S) solution (1 μl/ml Coronavirus 229E, 56°C heat-inactivated + 1 μM ATP or 1 μg/ml SARS-CoV-2 S protein + 1 μM ATP, respectively) for 1-2 min and then rinsed with H-PBS. Electrophysiological recordings were accomplished using an Axopatch 1-C amplifier, and pClamp10 software was used for data acquisition and analysis. Changes in peak conductance induced by the proteins were normalized to those recorded in H-PBS alone before exposure to the agents and expressed as fold changes.

##### Patient information

The institutional review board approved this non-interventional study of the ethical committee for research (CER) of the University of Paris Saclay (CER-Paris-Saclay-2020- 050), which conformed to the Declaration's principles of Helsinki. Accordingly, all participants have been written informed of the study and allowed to disengage. All patients examined were positive for SARS-CoV-2 RNA by RT-PCR at the hospital.

##### SARS-CoV-2 detection by RT-qPCR

500 μl of blood was processed for Reverse transcription and one-step quantitative polymerase chain reaction (RT-qPCR) as described (Real et al., 2020). Total RNA was extracted from 150 μl of PPP using NucleoSpin Dx Virus, Mini kit for CE-certified purification of viral RNA/DNA (Macherey-Nagel) according to the manufacturer’s recommendations. RT-qPCR was performed from 50 μl of eluted RNA, using TaqMan RNA-to-CT 1-step Kit (Applied Biosystems) and TaqMan 2019nCoV Assay Kit v1 (Thermo Scientific). Briefly, 4 targets are enrolled in our system. ORF1 gene (specifically the region encoding RNA-dependent RNA polymerase, RdRp), S gene (encoding Spike protein), and N gene (encoding nucleocapsid protein) aim at SARSCoV-2, human RNase P RPPH1 gene runs in a duplex with each SARS-CoV-2 assay, serving as an internal positive control. TaqMan 2019nCoV Control Kit v1 (Thermo Scientific) was used together to monitor assay-specific amplification. For each RT-qPCR reaction, a total volume of 20 μl comprises 5 μl of sample RNA, 10 μl of TaqMan PT-PCR Mix (2x), 0.5 μl of TaqMan RT Enzyme Mix (40x), 1 μl of TaqMan 2019nCoV assay, and 1 μl of RNase P Assay. RT-qPCR was performed on a LightCycler 480 Instrument II (ROCHE). The reaction was carried out at 48°C for 15 min, followed by an additional incubation at 95°C for 10 min, followed by 40 cycles of 95°C for 15 sec and 60°C for 1 min. Results were analyzed using the LightCycler 480 Software v1.5.

##### Bronchoalveolar lavage (BAL)

Broncho-alveolar lavages were collected as described (Delclaux et al., 1997) and processed within 3 h. BAL was passed through a 70-μm strainer and collected in a 50 ml tube. After the centrifugation of 500 g for 10 min, fluid was collected and aliquoted at 1 ml and stored at -80°C in a BSL3 laboratory. The BAL cell pellets were resuspended in 200 μl of the same individual BAL fluid, 20 μl of which were spotted and air-dried on SuperFrost Microscopic glass slides and kept in -80°C until use for *in situ* hybridization and confocal microscopy analyses as well as ATP/PGE_2_/IL-1β ELISA determinations. Samples were inactivated by heating at 65°C for 30 min as described (Loveday et al., 2021). Additional methods are presented and described in detail in the STAR Methods section.

##### Single-cell RNA sequencing nasal epithelia

Sequencing data was obtained from publicly available information generated by the scientific community and characterized through the COVID-19 Cell Atlas Project (https://www.covid19cellatlas.org/). Public datasets used in this publication were specifically generated by the Vieira Braga and Shalek labs and approved for both groups. Nasal epithelia were collected by nasopharyngeal brushes or swabs. The Vieira Braga dataset was collected from an upper airway nasal brush from patients not infected with COVID-19. The Shalek set was collected from nasal epithelial scrapes from uninfected and COVID-19 infected patients. As previously described, samples were collected from patients (Delorey et al., 2021; Ziegler et al., 2021). Representative cell populations were selected to display the relative expression of Panx-1 mRNA.

##### Immunofluorescence and confocal microscopy

Lung tissue sections were cut and processed, as described recently (Ganor et al., 2019; Valdebenito et al., 2021a, 2021b). Briefly, in addition to deparaffination, to eliminate or reduce autofluorescence from light sources in the green and red channel, tissues were incubated in Sudan Black and sodium borohydride to reduce autofluorescence and for antigen retrieval, as we described (Sanchez et al., 2009). The lung tissue sections were treated with the RNAscope Multiplex Fluorescent Reagent Kit v2 Assay protocol (ACDbio), following manufacturer instructions. The procedure includes multiple steps of sample pre-treatment, including RNAscope target retrieval reagent for 15 min, RNAscope protease plus for 15 min, hybridization (RNA probe V-nCOV2019-S-sense, specific for SARS-CoV-2), and signals development (TSA Plus cyanine 5 fluorophore, Opal 690), each step was followed by two wash steps with 1X wash buffer. Later the samples were incubated in a blocking solution for 2 h at room temperature and then incubated with primary antibodies overnight at 4°C. Cells were washed several times with PBS at room temperature and incubated with the appropriate secondary antibody for at least 2 h at room temperature, followed by another wash in PBS. Tissues were examined using an A1 Nikon confocal microscope with spectral detection (Nikon, Japan). Antibody specificity was confirmed by replacing the primary antibody with a non-specific myeloma protein or the same isotype or non-immune serum, as we described and suggested (Marakalala et al., 2016; Okafo et al., 2020; Valdebenito et al., 2020, 2021a, 2021b). Analysis of the 3D reconstruction and deconvolution was performed using NIS Elements (Nikon, Japan).

##### ATP assay

ATP concentration was determined using the ATPlite luminescence assay system (PerkinElmer, MA) by combining 100 μL of the sample with 100 μL of ATPlite reagent. Luminescence was measured using a PerkinElmer EnVision Multilabel Plate Reader. The extracellular concentration of ATP was determined by comparing sample luminescence to a standard curve generated using ATP standards provided by the manufacturer.

##### Analysis of IL-1β and PGE_2_ release

Tissue culture media and BAL were collected and inactivated at 65°C for 30 min as described for SARS (Rabenau et al., 2005a, 2005b, 2005c) and stored at −80°C. There were no freeze-thaw cycles before analysis. Samples were analyzed for IL-1β (Quantikine ELISA kit; R and D Systems, Minneapolis MN, USA) and PGE_2_ (Abcam, Cambridge, MA, USA) by enzyme-linked immunosorbent assay (ELISA) according to the manufacturer's instructions.

### Quantification and statistical analysis

The student’s two-tailed paired T-test was used to compare the different groups. A value of p<0.005 was considered significant. All statistical tests were implemented either in R (v4.0.2) or Prism (v6) software. Comparisons between cell-type proportions by disease group were tested using a Kruskal-Wallis test with FDR correction across all cell types, implemented in R using the Kruskal test and p.adjust functions. Post-tests for between-group pairwise comparisons used Dunn’s test. Spearman correlation was used where appropriate, implemented using the cor.test function in R. All testing for differential expression was implemented in R using either Seurat, scVelo, or DESeq2, and all results were FDR-corrected as noted in specific STAR Methods sections. P-values, n, and all summary statistics are provided in the results section, figure legends, figure panels, or supplementary tables.

## Data Availability

• Unique standardized data types were not generated. All other unique data will be shared upon reasonable request to the lead contact. • This study did not generate unique original code. • Any additional information required to re-analyze the data reported in this paper is available upon reasonable request to the lead contact.
