# Supplementary material for: Pannexin-1 channel opening is critical for COVID-19 pathogenesis
Source: iScience. 2021 Nov 19;24(12):103478. doi: 10.1016/j.isci.2021.103478 (PMC8603863; doi:10.1016/j.isci.2021.103478)
Supplement: Document S1. Figures S1 and S2 [file mmc1.pdf]

iScience, Volume *24*

Supplemental information

Pannexin-1 channel opening is critical for COVID-19 pathogenesis

Ross Luu, Silvana Valdebenito, Eliana Scemes, Antonio Cibelli, David C. Spray, Maximiliano Rovegno, Juan Tichauer, Andrea Cottignies-Calamarte, Arielle Rosenberg, Calude Capron, Sandrine Belouzard, Jean Dubuisson, Djillali Annane, Geoffroy Lorin de la Grandmaison, Elisabeth Cramer-Bordé, Morgane Bomsel, and Eliseo Eugenin

Supplemental Figures

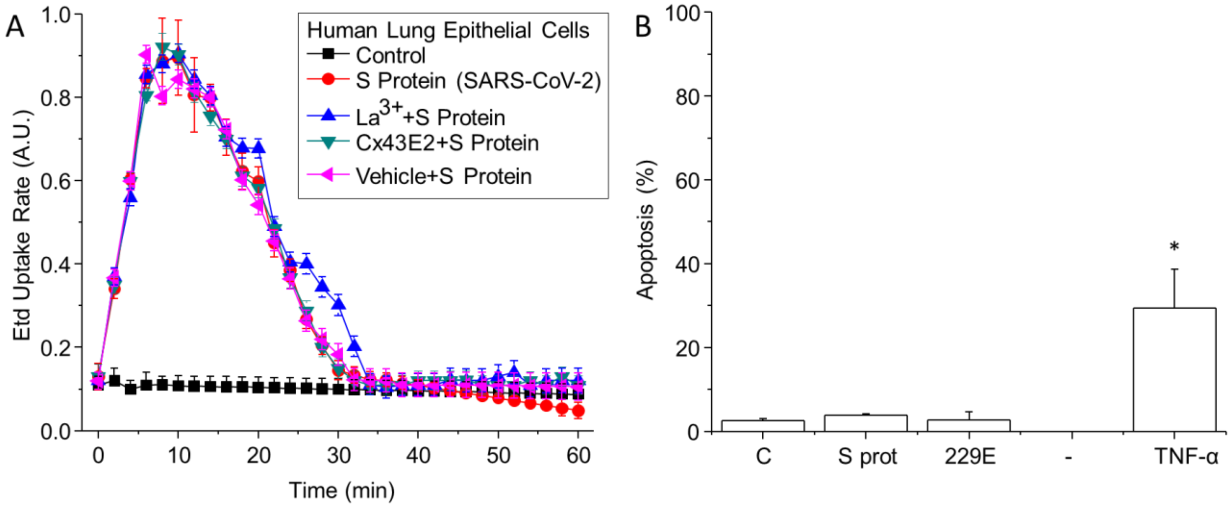

Figure S1. SARS-CoV-2 S protein-induced opening of a large channel is not mediated by Cx43 hemichannel opening. As demonstrated in Fig. 1, the opening of a large channel was mediated by Panx-1 channels. However, we have to discard the participation of Cx43 hemichannels. (A) Quantification of the time course of Etd uptake rate from human lung epithelial cells under control conditions (black square) or after SARS-CoV-2 S protein treatment (red circle) for 0 to 60 min. Human lung epithelial cells were pre-treated with Lanthanum, La^{3+}, a Cx43 hemichannel blocker (blue upward triangle), Cx43 extracellular loop peptide 2, Cx43E2, a Cx43 hemichannel opening (green downward triangle), and vehicle (Scram, pink leftward triangle) for 10 min before the treatment with SARS-CoV-2 S protein. Both Cx43 hemichannel openings did not prevent the channel opening induced by SARS-CoV-2 S protein. In Fig. 1 and 2, we identified that S protein results in Panx-1 channel opening. No significant differences were observed between S protein or Cx43 hemichannel treatments (p=0.406 to 0.5600, for all the points analyzed). Each value corresponds to the mean ± SD of the Etd intracellular intensity

present in at least 20 cells per time point (n=4). (B) Quantification of apoptosis after 1 h or 24 h post-treatment (24 h data is plotted). No significant apoptosis was detected due to the treatments, SARS-CoV-2 S protein or hCoV-229E (whole virus or S protein). As a positive control, TNF-α (100 ng/ml) was used. Each value corresponds to the mean ± SD of three independent experiments ($n=3$, $p\leq 0.0015$ compared to control untreated conditions). Related to Figure 1.

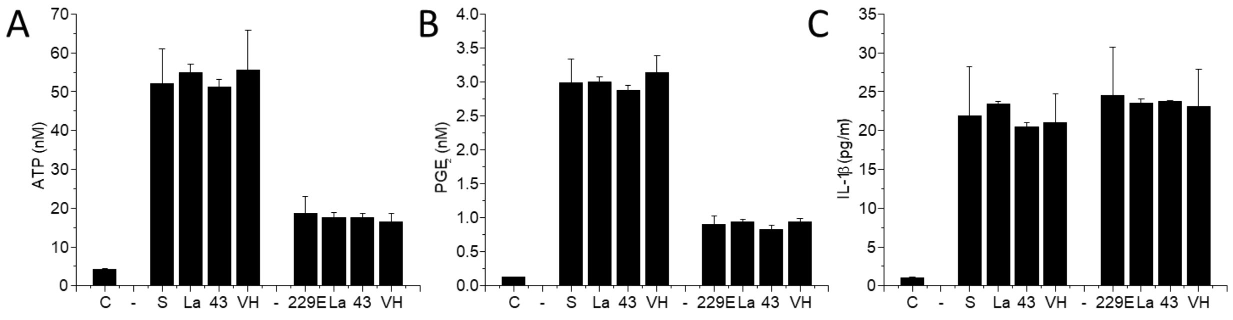

Figure S2. **Release of ATP, PGE_2, and IL-1β induced by SARS-CoV-2 S protein or hCoV-229E is not mediated by Cx43 hemichannel opening.** Upon treating primary lung epithelial cells with SARS-CoV-2 S protein (1 µg/mL) or hCoV-229E (0.1 MOI), media was collected after 1-, 6-, 12- and 24-h post-treatment to quantify ATP, PGE_2, and IL-1β. Data after 1-hour post-treatment are represented. (A) Determination of ATP secretion from primary human airway epithelial cells for the control (C) and after S protein (S Prot) or 229E virus (229E) treatment in the presence or absence of lanthanum (La) or Cx43E2 peptide (43), or vehicle (VH). Relative to the control (C), SARS-CoV-2 S protein and hCoV-229E treatment induced an ATP secretion independent of Cx43 hemichannel opening due that both inhibitors were ineffective in preventing ATP secretion. Each value corresponds to the mean ± SD (n=4). (B) Determination of PGE_2 secretion from primary human airway epithelial cells for the control (C) and after S protein (S Prot) or hCov-229E virus (229E) treatment in the presence or absence of Cx43 hemichannel blockers, lanthanum (La), or Cx43E2 peptide (43), did not affect PGE_2 secretion. Additionally, pre-treatment with the vehicle before treatment with S protein or 229E resulted in elevated concentrations of PGE_2 comparable to cells treated with S protein or 229E alone, respectively (*$p \leq 0.001$, n=4). Each value corresponds to the mean ± SD (n=4). **(C)** Determination of IL-1β secretion

from primary human airway epithelial cells for the control (C) and after S protein (S Prot) or hCoV-229E virus (229E) treatment in the presence or absence of Cx43 hemichannel blockers, lanthanum (La) or Cx43E2 peptide (43), or the vehicle (VH). Relative to the control (C), SARS-CoV-2 S protein and hCoV-229E treatment-induced IL-1β secretion was independent of Cx43 hemichannel opening. Each value corresponds to the mean ± SD (n=3). Thus, Cx43 hemichannels did not participate in ATP, PGE_2, and IL-1β secretion induced by both viruses, SARS-CoV-2 or hCoV-229E. Related to Figure 3.
